# New Hydrocarbon Degradation Pathways in the Microbial Metagenome from Brazilian Petroleum Reservoirs

**DOI:** 10.1371/journal.pone.0090087

**Published:** 2014-02-26

**Authors:** Isabel Natalia Sierra-García, Javier Correa Alvarez, Suzan Pantaroto de Vasconcellos, Anete Pereira de Souza, Eugenio Vaz dos Santos Neto, Valéria Maia de Oliveira

**Affiliations:** 1 Microbial Resources Division, Research Center for Chemistry, Biology and Agriculture (CPQBA), University of Campinas - UNICAMP, Campinas, Brazil; 2 Laboratory of Genomics and Expression, University of Campinas - UNICAMP, Campinas, Brazil; 3 Federal University of São Paulo - UNIFESP, São Paulo, Brazil; 4 Center of Molecular Biology and Genetic Engineering – CBMEG/UNICAMP, Rio de Janeiro, Brazil; 5 PETROBRAS/R&D Center, Rio de Janeiro, Brazil; Missouri University of Science and Technology, United States of America

## Abstract

Current knowledge of the microbial diversity and metabolic pathways involved in hydrocarbon degradation in petroleum reservoirs is still limited, mostly due to the difficulty in recovering the complex community from such an extreme environment. Metagenomics is a valuable tool to investigate the genetic and functional diversity of previously uncultured microorganisms in natural environments. Using a function-driven metagenomic approach, we investigated the metabolic abilities of microbial communities in oil reservoirs. Here, we describe novel functional metabolic pathways involved in the biodegradation of aromatic compounds in a metagenomic library obtained from an oil reservoir. Although many of the deduced proteins shared homology with known enzymes of different well-described aerobic and anaerobic catabolic pathways, the metagenomic fragments did not contain the complete clusters known to be involved in hydrocarbon degradation. Instead, the metagenomic fragments comprised genes belonging to different pathways, showing novel gene arrangements. These results reinforce the potential of the metagenomic approach for the identification and elucidation of new genes and pathways in poorly studied environments and contribute to a broader perspective on the hydrocarbon degradation processes in petroleum reservoirs.

## Introduction

Several studies have shown the ability of aerobic and anaerobic bacteria to degrade hydrocarbon compounds [1]. Traditional culturing techniques have been used to obtain valuable information on microbial interactions with hydrocarbons in the environment and have allowed the identification of many types of bacteria capable of utilizing hydrocarbons and the operons encoding these degradation pathways. However, only a small fraction of the microbial diversity in nature (1–10%) can be grown in the laboratory [2]–[4]. Therefore, the ecological functions of the majority of microorganisms in nature and their potential applications in biotechnology are still obscure [5].

Oil reservoirs constitute deep geological environments where microbial activities over millions of years have caused significant biodegradation of crude oils worldwide. Understanding the microbial processes, in situ microorganisms and factors governing the biodegradation of crude oil hydrocarbons in vast oil reservoirs remains a challenge [6], not only because of the complex microbiological sampling and the inaccessibility of petroleum reservoirs, but also because of the repeated isolation of the same species when cultivation techniques are employed [7].

Metagenomics is a molecular tool that overcomes the limitations imposed by the classical approach, enabling a broader perspective of the taxonomic and functional variety of environmental microorganisms and access to their metabolic potential [8]. The number of metagenomic projects has exploded in recent years, and hundreds of environmental samples have been unraveled by shotgun sequencing [9]. However, this is the first report of a metagenomic library obtained from petroleum microbial enrichments in the current Genomes Online Database (GOLD; http://www.genomesonline.org; accessed August, 2012), which lists 335 completed or ongoing metagenome projects.

Recently, we used a function-driven metagenomic approach to identify diverse and potentially novel hydrocarbon biodegraders in petroleum reservoirs [10]. A fosmid library was constructed using the metagenomic DNA prepared from aerobic and anaerobic enrichments of a biodegraded petroleum sample. Hexadecane was used to screen the library for hydrocarbon-degrading fosmid clones. Seventy-two of the 5,000 fosmid clones screened were able to grow using hexadecane as the carbon source. Of these 72 clones, five were able to degrade >70% of the hexadecane in chromatographic assays (GC-MS). In this study, the aromatic compound degradation ability of these 5 clones was evaluated and the inserts of these clones were fully sequenced, which provided new insights into the sequence diversity of the hydrocarbon degradation proteins and revealed novel gene arrangements.

## Materials and Methods

### Aromatic Compound Degradation Ability

Five fosmid clones from a metagenomic library degrading over 70% of the hexadecane [10] were subjected to biodegradation assays using naphthalene and phenanthrene. The assays were performed as previously described by Vasconcellos et al. [10]. The clones were incubated in 50 ml of mineral medium (NaCl, 14 g/l; KHPO_4_, 2.8 g/l; KH_2_PO_4_, 2.8 g/l; (NH_4_)_2_SO_4_, 2.8 g/l; MgSO_4_. H_2_O, 0.56 g/l; NaNO_3_, 8.4 g/l) containing chloramphenicol (12.5 µl/ml), vitamin solution (50 µl) [11], 0.1% hydrocarbon (according to the substrate:inoculum ratio described by Vasconcellos et al. 2010) as the sole carbon source and 5 ml of the cell suspension (10^8^ CFU/ml) of the fosmid clone culture. The assays were run in triplicate and monitored using GC–MS for 10 and 14 days for naphthalene and phenanthrene, respectively. In addition, biodegradation negative controls were performed as follows: C1 = culture medium + hydrocarbon; C2 = culture medium + hydrocarbon + *E. coli* host cells. Doubly distilled hexane was used as the extraction solvent for the organic phase in the chromatographic analysis. Nonadecane solution (0.5 mg/ml) was used as the internal standard. The GC–MS assays were performed according to Vasconcellos et al. [12]. The extent of biodegradation was calculated from the chromatographic data according to Vasconcellos et al. [12].

### Extraction of Fosmid DNA and Sequencing

The clones were subjected to complete insert sequencing, as follows. First, clones 1A, 2B, 3B, 10A and 6A were grown individually in Erlenmeyer flasks containing 150 mL of LB medium and chloramphenicol (12.5 µg/mL) for 17 h at 37°C and 180 rpm. Subsequently, the fosmid DNA was isolated using the Invisorb Plasmid Maxi Kit (Invitek, Berlin, Germany), according to the manufacturer’s protocol for large constructs. Finally, the purified DNA from fosmid clone 2B was sent to Macrogen Inc. (Seoul, Korea) for shotgun library construction and sequencing with 8X coverage in a Sanger sequencer. The remaining four clones (1A, 3B, 6A and 10A) were sequenced at UNC (Chapel Hill, NC) using an Illumina Hiseq2000 Single-end 50 bp, and library construction was performed according to the manufacturer’s instructions.

### Bioinformatic Analyses of the Fosmid–derived ORFs

The fosmid DNA reads generated by Illumina were assembled into a unique contiguous fragment (contig) using the Velvet algorithm [13]. Sequences obtained for fosmid clone 2B using Sanger sequencing were assembled into a single contig at Macrogen Inc. The open reading frames (ORFs) in all fosmids were identified using several tools available for gene prediction in prokaryotes through heuristic approaches: Metagene [14] http://metagene.cb.k.u-tokyo.ac.jp/) and MetaGeneMark [15] designed for metagenomic sequences, GLIMMER 3.02 [16], [17] and FGENESB (http://linux1.softberry.com/berry.phtml). The predicted ORFs were assigned putative functions based on BLASTp searches against protein sequences in the NCBI and UniProtKB databases using an E-value threshold of >10^−3^, and protein family databases using InterProScan (encompassing the PROSITE, PRINTS, Pfam, ProDom, SMART and TIGRFAMMs databases) [18]. Cognitor [19] was used to assign each ORF to functional categories called Clusters of Orthologous Groups (COG). The final ORF annotations were performed manually using the criteria proposed by Liu et al. [20] and were viewed and edited with the Artemis 12.0 software [21], which was also used to calculate the GC content (G+C%).

Putative ribosomal binding sites were identified using RBSFINDER [22], and the presence of bacterial promoters and transfer RNA genes was predicted using the programs BPROM (http://linux1.softberry.com/berry.phtml) and tRNAscan-SE [23], respectively. Putative metabolic pathways were analyzed using MetaCyc [24] and the KEGG database [25]. Automatic annotation was also performed using The Rapid Annotation using Subsystem Technology –RAST Server version 4.0 [26]. Additionally, TMHMM was used to identify transmembrane domains [27].

### Phylogenetic Affiliations of the Metagenomic Sequences

Two methods were used to establish the phylogenetic affiliations of the fosmid inserts. The first approach was based on the oligonucleotide composition calculated using the PhyloPythia software [28], which is a phylogenetic classifier that uses a multi-class support vector machine (SVM) for the taxonomic assignment of variable-length metagenome sequence fragments based on their oligonucleotide compositions.

The second approach used to classify the metagenomic sequence fragments was based on a search for “marker genes”, which are phylogenetic anchors used to identify the source organism of a given fragment. In the absence of the 16S ribosomal RNA phylogenetic marker, other genes, particularly those involved in housekeeping functions (COG J, K, and L categories) were used. Housekeeping genes have been studied extensively [29] and used for reconstruction of organismal evolution because they are relatively ubiquitous and rarely affected by horizontal gene transfer [30]. For protein phylogenies, the closest orthologs were identified using the BLASTp tool (NCBI), the amino acid sequences were aligned using the CLUSTAL W program [31], and the phylogenetic trees were constructed using the *Neighbor-Joining* distance algorithm [32] with bootstrap values calculated from 1000 replicate runs using the MEGA v.5.0 software [33].

### Data Accessibility

The metagenome project was deposited at the Genomes On Line Database [34] and the complete fosmid sequences for clones 1A, 2B, 3B, 6G and 10A were submitted to GenBank under the accession numbers KC130084, KC157637, KC157638, KC157639 and KC157640, respectively.

## Results and Discussion

### Hydrocarbon Degradation Analysis

Five fosmid clones FOS1A, FOS2B, FOS3B, FOS6A and FOS10A were analyzed for aromatic hydrocarbon degradation using GC-MS. Except for FOS1A, the clones were able to degrade aromatic hydrocarbons at low or high levels after 10–14 days of monitoring (Table 1). The biodegradation results of the negative controls allowed us to trust the degradation percentages observed for the fosmid clones (Table 1). There is only one previous study on aromatic hydrocarbon degradation in clones (using a heterologous expression system) that can be used to evaluate our results (Vasconcellos et al. [10]); in that study, degradation of more than 70% of the hexadecane by the metagenomic clones was detected after 28 days. However, it is important to highlight that the hydrocarbon used by these authors (hexadecane) belongs to a structurally different class; aromatic hydrocarbons require longer periods of time to be degraded and become bioavailable and, consequently, extended periods of incubation.

**Table 1:** Hydrocarbon biodegradation by the metagenomic fosmid clones.

	Biodegradation extent (%)	
	Phenanthrene	Naphthalene
**FOS 1A**	5	Nd^a^
**FOS 2B**	44	7
**FOS 3B**	21	4
**FOS 6A**	15	78
**FOS 10A**	49	Nd^a^
**C1**	Nd^a^	Nd^a^
**C2**	Nd^a^	Nd^a^

a Not detected.

Therefore, the observed extent of biodegradation should be analyzed considering that these results refer to the activity detected in a heterologous system. In fact, function-based screening of metagenomic libraries for the degradation of xenobiotics is considered problematic because of insufficient and biased gene expression in *Escherichia coli* [35].

### General Features of the Metagenomic Fosmid Inserts

**Figure 1:**
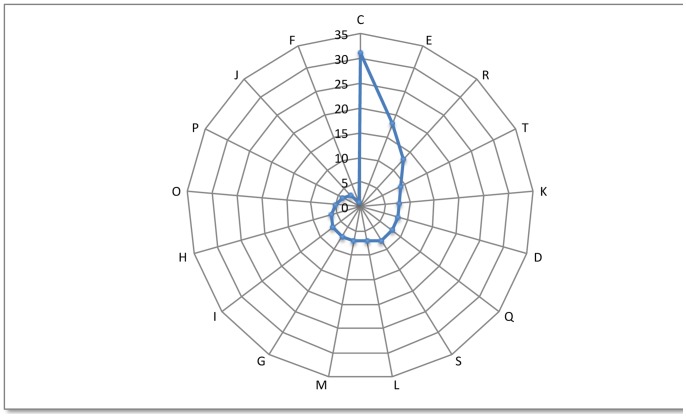
Functional assignment of the metagenomic fosmid clones using the COG database. (C) Energy production and conversion; (E) Amino acid transport and metabolism; (R) General function prediction only. (T) Signal transduction mechanisms; (K) Transcription; (D) Cell cycle control, mitosis, and meiosis; (Q) Secondary metabolites biosynthesis, transport and catabolism; (S) Function unknown; (L) Replication, recombination, and repair; (M) Cell wall/membrane biogenesis; (G) Carbohydrate transport and metabolism; (I) Lipid transport and metabolism; (H) Coenzyme transport and metabolism; (O) Post-translational modification, protein turnover, chaperones; (P) Inorganic ion transport and metabolism; (J) Translation; (F) Nucleotide transport and metabolism.

Shotgun sequencing was performed on the five fosmids and a single contig was assembled for each fosmid. The general features of the fosmid inserts are summarized in Table 2. The insert fragments ranged from 30 to 42 kb in length and from 63.4 to 66% in G+C content. In total, 181 ORFs were identified; 10% of these ORFs were hypothetical with unknown function, 83% were assigned to clusters of orthologous groups of proteins (COGs) and 17% had no related COG group. The distribution pattern of the COG-assigned proteins (Fig. 1, Supplemental Table S1) highlights the dominance of categories C (21%) and E (12%). The predominance of proteins belonging to COG category C (energy production and conversion) could indicate the potential of the corresponding fosmid clones to obtain energy from different conditions or substrates. Predominance of proteins belonging to COG category E (amino acid transport and metabolism), particularly the components of the ABC-type transport system, were repeatedly detected in all fosmids. The ABC-type transporter genes are frequently present in gene clusters associated with aromatic compound catabolism [36]–[38].

**Table 2:** Characterization of the fosmid inserts of the metagenomic clones: insert size, G+C content and number of ORFs.

	FOS1A	FOS2B	FOS3B	FOS6A	FOS10A
**Fosmid insert size (kb)**	34.8	37.4	42.2	33.8	30.3
**G+C-content (%)**	65,57	65,32	65,29	63,42	66,01
**Number of ORFs**	32	40	46	31	32
**Number of hypothetical** **proteins**	4	3	5	1	5
**Number of ORFs with** **COG assignment**	28	36	41	28	17
**Classification result** **(Phylum/Class)**	Proteobacteria/B-proteobacteria	Proteobacteria/B-proteobacteria	Proteobacteria/B-proteobacteria	Proteobacteria/B-proteobacteria	Proteobacteria/G-proteobacteria

### Gene Order and Regions of Synteny between Fosmid Clones

The full-length fosmid insert sequences were subjected to gene prediction and manual annotation. Detailed information on the putative functions of the 181 ORFs in each metagenomic fragment and references to the best BLASTp hits used for annotation are shown in Tables S2 to S6 in the supplemental material.

**Figure 2:**
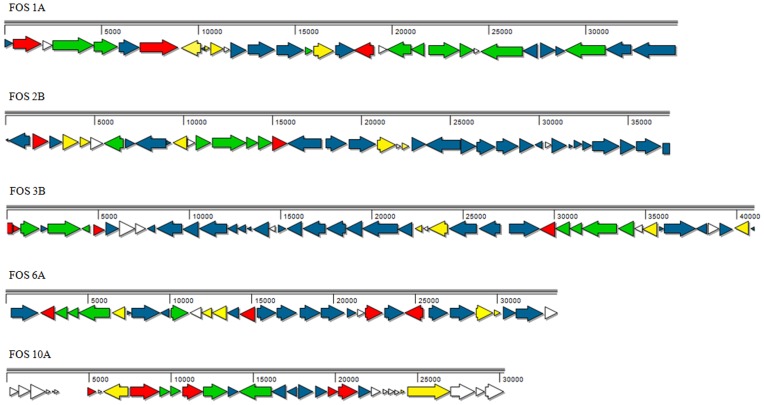
Schematic representation of the sequence annotations of the entire insert in five metagenomic fosmid clones. The identified open reading frames (ORFs) are shown in arrows, and the start and stop codons and the coding direction are indicated. The ORFs are color coded according to their functional category assigned by COG (Clusters of Orthologous Groups of proteins). Blue arrows: metabolism; green arrows: cellular processes and signaling; red arrows: information storage and processing; yellow arrows: poorly characterized; white arrows: not in COG. Further details on the ascribed putative function for each ORF are indicated in Supplementary tables S2 to S6.

**Figure 3:**
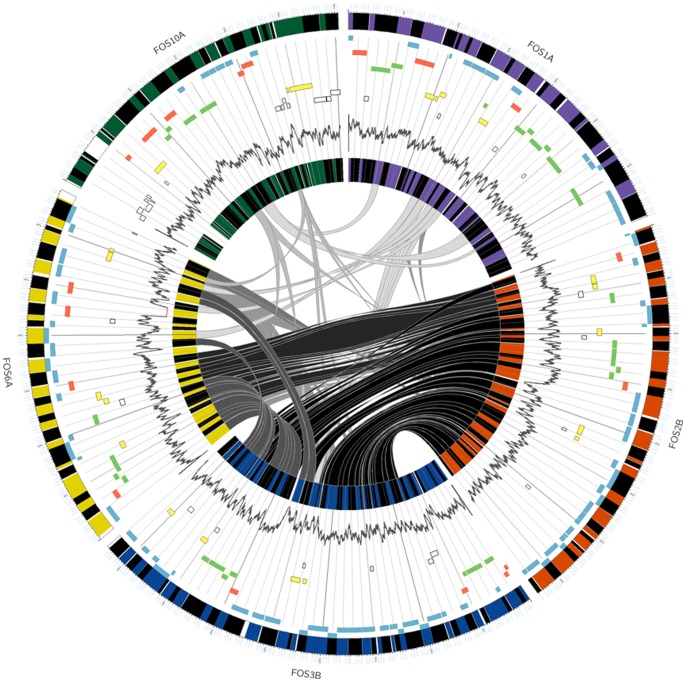
Circular representation of the five annotated fosmid clones and synteny between them. The outermost ring displays the different fosmids in different colors (dark green, purple, orange, dark blue and dark yellow). Annotated ORFs are shown in blocks (black is used to distinguish consecutive ORFs in the same fosmid). The connecting lines inside the circle join syntenic regions. The gray scale indicates the levels of synteny detected between the fosmids; the darkness is proportional to the number of genes in the two different fosmids (i.e., FOS 3B and FOS 2B showed more synteny, followed by FOS 3B and FOS 6A, and so on). Universal clusters of orthologous groups are shown in different colors (rings 2, 3, 4, 5); light blue: metabolism, light green: cellular processes and signaling, red: information storage and processing, light yellow: poorly characterized; white: not in COG. The innermost ring shows the G+C content (%). This figure was created using the Circos software (Krzywinski et al., 2009).

The genetic organization of all ORFs, their directions and COG classifications in each fosmid insert are shown in Figure 2. The fosmid clones had common genes and gene clusters of variable length. Two clones, FOS2B and FOS3B, showed the largest region of synteny; however, fosmid FOS3B was larger and encoded an additional cluster of genes involved in arsenate resistance (Figure 3). Clone FOS6A was similar to FOS2B and FOS3B, with the same genes encoding proteins related to the anaerobic and aerobic degradation of aromatic compounds, genes coding for other metabolic functions and regulatory genes.

The genetic organization of clone FOS10A was different from that of the other clones and included an uncommon region where no coding regions were identified. Curiously, this noncoding region was flanked by sequences designated as “clustered regulatory interspaced short palindromic repeats”- CRISPR, which describes a class of DNA repeats found in nearly half of all bacterial and archaeal genomes [39]. The CRISPR system is a prokaryotic immune system that provides protection against infection by mobile DNA elements, including viruses. The DNA repeats are transcribed and processed into small RNAs that confer resistance to phages (i.e., viruses that infect bacteria). Immunity is acquired by the capture of short viral DNA sequences known as “protospacers”, which are incorporated into the host genome, flanked by the CRISPR repeat sequences, and subsequently termed “spacers” [40]. Consistently, the noncoding region of 1745 nucleotides observed immediately adjacent to the CRISPR sequences was identified as a spacer.

### Functional Analysis of the Metagenomic Fosmid Sequences

**Figure 4:**
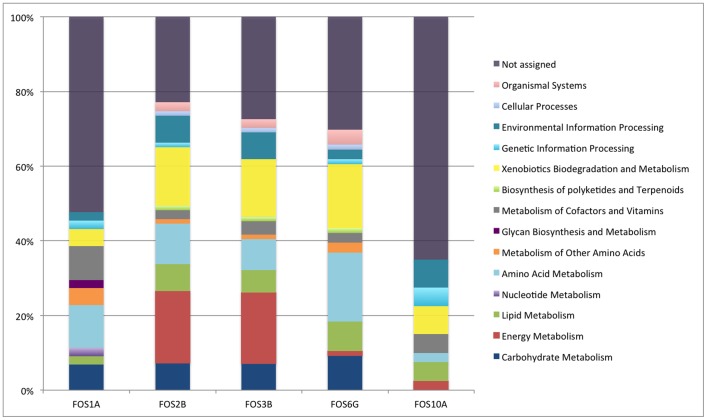
KEGG-based functional assignment of the sequences from the metagenomic fosmid clones.

The sequences were functionally annotated based on the RAST platform using the KEGG database. The functional analysis allowed the classification of the sequences into several KEGG categories; the majority of the sequences were related to the xenobiotic, amino acid, energy and carbohydrate metabolism subcategories (Figure 4). Although a high percentage of sequences could not be assigned to any KEGG category, several genes encoding multiple hydrocarbon degradation pathways were identified in all fosmid clones using comparative tools in the RAST platform. The numbers of sequences related to the most representative KEGG categories in each fosmid clone are listed in Table 3.

**Table 3:** Number of sequences of the metagenomic fosmid clones associated with specific functions in KEGG pathways.

KEEG category	FOS1A	FOS2B	FOS3B	FOS6A	FOS10A
***Biodegradation of xenobiotics***					
Biphenyl degradation	0	1	1	1	0
Bisphenol A degradation	1	0	0	0	0
Geraniol degradation	0	0	0	0	1
3-Chloroacrylic acid degradation	2	4	3	3	0
1,2-Dichloroethane degradation	1	2	1	2	1
Naphthalene and anthracene degradation	1	1	1	1	0
Ethylbenzene degradation	0	0	0	0	1
Fluorene degradation	0	1	1	1	0
Benzoate degradation via CoA ligation	0	1	0	1	2
Benzoate degradation via hydroxylation	0	1	1	1	0
1- and 2-Methylnaphthalene degradation	1	2	2	2	1
Tetrachloroethane degradation	1	0	0	0	0
Trinitrotoluene degradation	1	0	0	0	0
Gamma-Hexachlorocyclohexane degradation		1	1	1	
Caprolactam degradation			1		
Metabolism of xenobiotics by cytochrome P450	0	1	1	1	0
Drug metabolism – cytochrome P450	0	1	1	1	0
1,1,1-Tricloro-2,2-bis(4-chlorophenyl)ethane (DDT) degradation	0	1	1	1	0
Ubiquinone and other terpenoid-quinone biosynthesis	1	1	2	1	0
***Carbohydrate metabolism***					
Glycolysis/Gluconeogenesis	2	2	2	2	0
Ascorbate and aldarate metabolism	1	2	2	2	0
Pyruvate metabolism	1	2	2	2	1
Propanoate metabolism	1	1	1	1	1
Butanoate metabolism	3	1	1	2	2
**Amino acid metabolism**					
Tyrosine metabolism	3	2	3	3	1
Alanine, aspartate and glutamate metabolism	2	0	0	3	0
Arginine and proline metabolism	4	1	1	4	0
Glycine, serine and threonine metabolism	1	1	1	1	0
Histidine metabolism	2	1	2	1	1
Beta-Alanine metabolism	2	1	1	1	0
Tryptophan metabolism	2	3	3	3	1
**Lipid metabolism**					
Fatty acid metabolism	2	4	3	4	1

**Figure 5:**
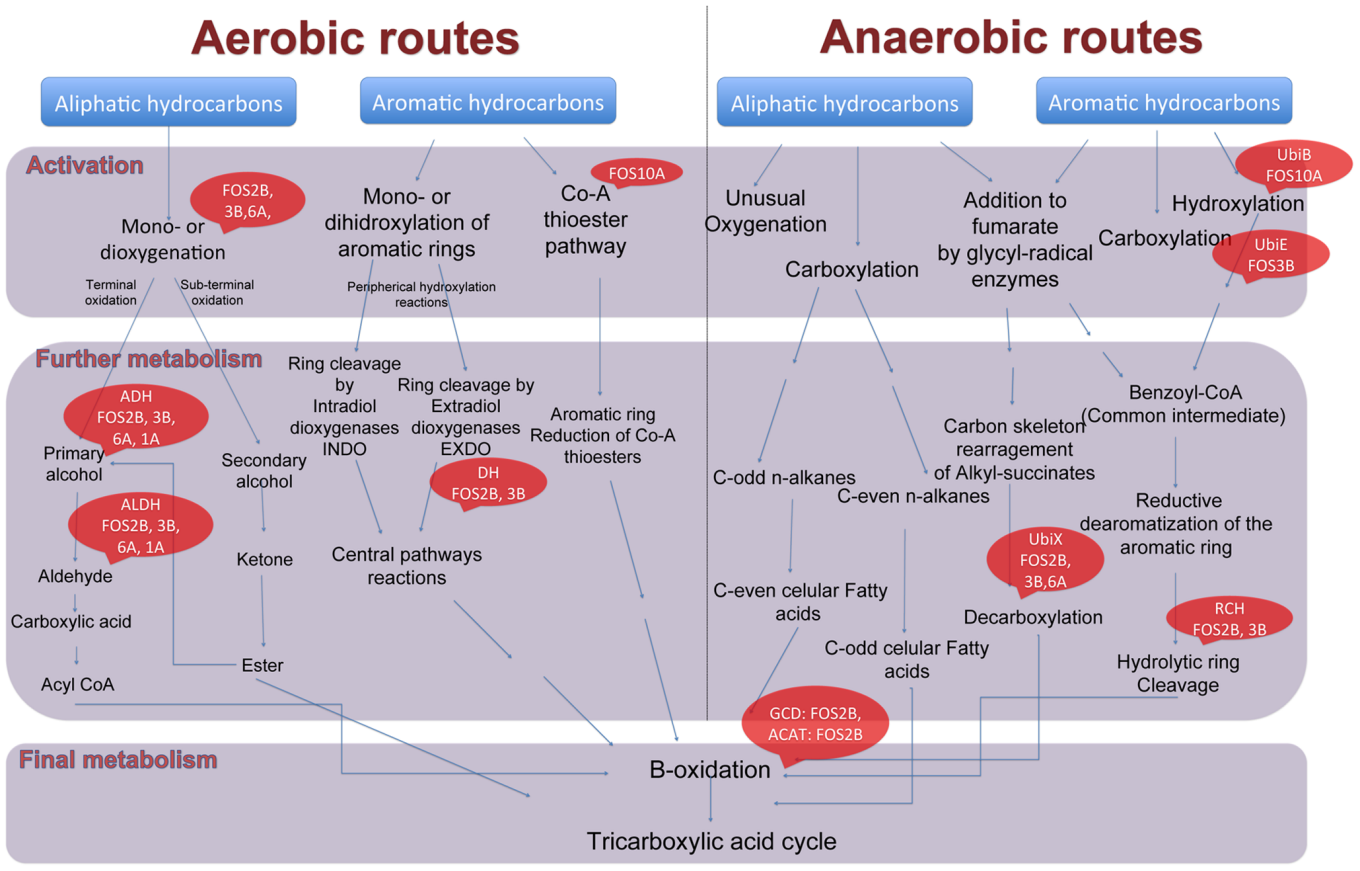
Comprehensive map of biochemical pathways for aerobic and anaerobic bacterial degradation of hydrocarbon compounds. Red bubbles show which genes of these routes are present in one or more fosmids. Two consecutive arrows represent more than one reaction. (UbiB) Ubiquinone biosynthesis protein UbiB; (UbiE) Ubiquinone biosynthesis protein C-methyltransferase UbiE; (ADH) Alcohol dehydrogenase; (ALDH) Aldehyde dehydrogenase: (DH) Dienelactone hydrolase; (UbiX) 3-octoprenyl-4-hydroxybenzoate; (RCH) Ring cleavage hydrolase; (GCD) Glutaryl-CoA dehydrogenase; (ACAT) Acetyl-CoA acyltransferase. Figure modified from [85].

None of the fosmid clones had a complete map or gene cluster reported to be involved in the catabolism of hydrocarbon compounds in the hydrocarbon degrading bacteria. We found that the metagenomic fragments contained subsets of genes belonging to different pathways previously described in other assemblages for anaerobic and aerobic degradation of different aromatic and aliphatic compounds (Figure 5). The degradation of environmental aromatic compounds through the concerted action of various fragmented pathways has been previously observed using metagenomic data [41], [42] and in isolated strains [43].

### Overview of Putative Genes Involved with Aromatic Hydrocarbon Degradation

The fosmids with the highest naphthalene- and phenanthrene-degradation abilities (FOS2B, 6A and 10A) were screened for the presence of specific ORFs in the Naphthalene and Anthracene pathway using KEGG. The only enzyme of this pathway found was haloalkane dehalogenase (EC 3.8.1.5– Naphthalene and Anthracene pathway), identified in all fosmids except FOS10A.

Nonetheless, ORFs related to hydrocarbon activation and/or generic degradation pathways were found in all fosmids (Figure 5). The fosmids were presumed to encode activation mechanisms based on aerobic reactions. Sequences related to the cytochrome P450 proteins were found in three metagenomic fosmid clones (FOS2B, FOS3B and FOS6A). The P450-type hydroxylases are commonly used for the aerobic activation of alkanes with medium chain lengths in bacteria [44], [45]. Furthermore, P450-monooxigenases are involved in aromatic hydrocarbon activation in filamentous fungi and mammals [46]. Sequences related to another mechanism of aromatic hydrocarbon activation were identified in the fosmids FOS2B, 3B and 6A, suggesting a hydrocarbon activation via CoA thioesterification (Figure 5).

Genes related to anaerobic mechanisms for hydrocarbon activation were also identified in the fosmid 10A. The protein encoded by ORF 9 might activate hydrocarbons via a hydroxylation step in the ubiquinone biosynthesis pathway, leading to the production of the corresponding catechol [47]. Although the most common activation mechanism under anaerobic conditions in phylogenetically diverse microbial populations is the addition of fumarate to aromatic hydrocarbons via benzylsuccinate synthase (BssABCD) [38], [48], the absence of *bss* genes has also been reported for *Dechloromonas aromatica* str. RCB and *Azoarcus* BH72, which are anaerobic degraders of diverse aromatic compounds [49].

Once aromatic hydrocarbons are activated, further catabolism involves a wide variety of peripheral pathways acting on a large diversity of substrates into the key central common intermediates. Genes encoding these additional steps for the conversion of hydrocarbons to more easily biodegradable intermediates were found in the fosmids and are described below.

Apparently, specific genes for naphthalene and phenanthrene degradation were not found in the fosmids; thus, the observed degradation of phenanthrene by the fosmids 10A (49%) and 2B (44%) and naphthalene by the fosmid 6A (78%) could be explained by the action of a hitherto unknown protein encoded by the metagenomic inserts and/or the combined action of generic (non-specific) enzymes involved in the hydrocarbon degradation found in these fosmids (Figure 5).

### Organization of the Aerobic Hydrocarbon Degradation Genes in the Metagenomic Fragments

ORFs 4 and 14 in fosmids FOS2B and 3B, respectively, encode the enzyme dienelactone hydrolase (DLH). Dienelactone hydrolases have an α/β fold and play a fundamental role in the degradation of chlorocatechols, which are central intermediates in the degradation of chloroaromatics such as (chloro)benzoates, (chloro)naphthalenes, (chloro)salicylates, (chloro)benzenes and (chloro)phenols [50]. The chlorocatechol pathway involves the cleavage of the aromatic ring by four enzymes: chlorocatechol 1,2-dioxygenase, chloromuconate cycloisomerase, dienelactone hydrolase (DLH) and maleylacetate reductase (MAR) [51]. Pieper [52] identified that the genes encoding the chlorocatechol pathway enzymes are present in clusters and that the structures of the corresponding operons are highly conserved despite the geographically distinct origins of the bacteria or differences in their phylogeny. However, the DLH coding sequences in fosmids FOS2B and FOS3B were not flanked by sequences encoding other enzymes of the chlorocatechol degradation pathway, and no similar clusters were identified previously. Nonetheless, regulatory elements (e.g., the LysR family) and genes encoding transport proteins were detected upstream and downstream of the catabolic genes, respectively, in these fosmids, consistent with Pieper [52].

All fosmids except FOS10A (FOS1A, FOS2B, FOS3B and FOS6A) had genes encoding enzymes for the oxidation of alcohols (alcohol dehydrogenases) and aldehydes (aldehyde dehydrogenases), which constitute the second and third steps in the alkane oxidation pathway, respectively. The substrate specificity of alcohol dehydrogenases (ADHs) is not restricted to aliphatic alcohols; xenobiotic aromatic and alicyclic hydroxyls are also metabolized through similar pathways, highlighting the physiological importance of this enzyme system [53]. Aldehyde dehydrogenases (ALDHs) are widely distributed in living organisms and are involved in the detoxification of the toxic aldehydes produced by several cellular metabolic pathways, being recognized as one of the essential enzymes for the degradation of many hydrocarbon compounds.

The genes encoding ADHs and ALDHs are present in the best-characterized system of alkane degradation, the OCT plasmid of *Pseudomonas putida* GPo1. This plasmid encodes the entire pathway for n-alkane degradation and conversion to fatty acids. The general organization of the operon *alk*BFGHJKL in the OCT plasmid of *P. putida* GPo1 includes ADH and ALDH genes, sequences for the non-heme integral membrane alkane monooxygenase (AlkB) and other enzymes involved in additional steps. Similarly, the regions flanking the ADH and ALDH genes in the metagenomic fosmids FOS2B, FOS3B and FOS6A contain sequences for P450 enzymes. However, the presence of genes encoding ADHs and ALDHs in fosmid FOS1A could be associated with the presence of a novel alkane oxidation system, which has not been described so far, or could be explained by the previously reported redundancy of ADH and ALDHs in genomes [54]. Notably, the ADH and ALDH genes were also detected in an anaerobic methanogenic enrichment culture by Head et al. [55]. Those authors considered the possible association of the ADH and ALDH genes with an unspecified anaerobic hydroxylation reaction for the initial steps of alkane degradation.

### Organization of the Anaerobic Aromatic Degradation Genes in the Metagenomic Fragments

After aromatic compounds are activated, the subsequent degradation steps involve reductive de-aromatization and hydrolytic ring cleavage [56]. During the anaerobic degradation of activated benzene (benzoyl-CoA), the first step is the aromatic reduction of the benzene ring by enzyme benzoyl-CoA reductase, which catalyzes the de-aromatization of a diene benzoyl-CoA [57], [58]. The second step is a modified β-oxidation pathway involving the addition of the (di)enoyl-CoA, resulting in the cleavage of the ring. The last step is the conventional β-oxidation to 3-acetyl-CoA and CO_2_ [59], [60]. ORFs 12 and 39 of the fosmids FOS2B and 3B, respectively, were annotated as encoding a benzoate degradation ring-cleavage hydrolase, which functions in the modified β-oxidation step in the pathway described above [61].

Remarkably, there were no homologs of genes encoding the four subunits of benzoyl-CoA reductase, which suggests the involvement of a different enzymatic mechanism for the de-aromatization of the benzene ring. In the strictly anaerobic microorganism *Geobacillus metallireducens* [61], although the activation and modified β-oxidation steps of benzoate degradation are highly similar to other anaerobic organisms, the gene encoding the benzoyl-CoA reductase has not yet been identified. These data suggest that the processes for de-aromatization in *G. metallireducens* and most likely in fosmid 2B and 3B remain unknown.

Apart from the aerobic and anaerobic strategies for degradation, extensive genomic and biochemical studies have led to the identification of a hybrid pathway, termed the benzoate oxidation (box) pathway. The box pathway integrates the traditional anaerobic step of CoA ligation with an aerobic step that introduces oxygen to activate the aromatic ring for cleavage [62]. This hybrid strategy was first described for the catabolism of benzoate [63], but it has been recently described for the mineralization of other aromatic compounds [64].

In fact, the use of hybrid pathways (with the formation of CoA thioester intermediates) as a degradation mechanism by microorganisms in petroleum environments may help them to survive, allowing flexibility and rapid adaptation to fluctuating oxygen levels because both oxic and anoxic situations require CoA thioester substrates.

All fosmids except for FOS1A contained genes encoding enzymes for ubiquinone biosynthesis. The reactions that occur during ubiquinone biosynthesis, such as decarboxylation, methylation and hydroxylation, resemble the reactions carried out during bacterial catabolism of aromatic compounds [47]. Some reaction steps of the ubiquinone synthesis were encoded in several fosmids, and some others were unique to a specific fosmid. The third step in the biosynthesis of ubiquinone is a decarboxylation event catalyzed by 3-octaprenyl-4-hydroxybenzoate carboxy-lyase (UbiX). Enzymes related to UbiX are involved in phenol or hydroxybenzoate metabolism in strict anaerobes [59] and were encoded by ORF 9 of FOS2B, ORF 42 of FOS3B and ORF 9 of FOS6A. The *ubi*X gene is involved in anaerobic catabolism by *T. aromatica* [65], in the differential expression of the NaphS2 strain (Deltaproteobacteria) when grown in naphthalene [66], and it was located downstream of the benzoate degradation gene in a metagenomic fosmid clone described by Kube et al. [67], suggesting the ability of the native organism to use phenol anaerobically.

Other reactions in the biosynthesis of ubiquinone involve hydroxylations and methylations of the aromatic ring. Hydroxylation reactions are performed by the *ubi*B, *ubi*H and *ubi*F gene products, and methylation reactions are performed by the *ubi*E and *ubi*G gene products. In this study, *ubi*B and *ubi*E genes were detected in FOS10A (ORF 9) and FOS3B (ORF 8), respectively.

### ORFs Involved in Other Metabolic Characteristics

Elements of the LysR family are known regulators of aerobic pathways that act via catechol or protocatechuate pathways [60] and were found in FOS2B and FOS6A in association with acyl-CoA dehydrogenases. Other regulatory genes encoded in the metagenomic fragments belong to various families of bacterial transcriptional regulators, such as the two-component systems (in FOS1A), which are known to regulate aerobic and anaerobic toluene metabolism pathways in *P. putida* and *T. aromatica*, and sigma 54-dependent regulators, which are known to regulate aerobic and anaerobic phenol degradation in *Pseudomonas* sp. str. CF600 and *T. aromatic*. Nonetheless, as noted by Fuchs and co-workers [68], the control of aromatic-compound degradation varies widely among the different species of aerobic and anaerobic bacteria.

All components of ATP synthase were encoded in the metagenomic inserts of FOS2B and FOS3B and were organized in a single operon. The presence of genes encoding ATP synthesis and hydrocarbon degradation could be due to the energetic dependence of certain anaerobic respiration pathways, for example, the ATP-dependent ring reduction of CoA thioesters in facultative anaerobes [68].

FOS1A, FOS6A and FOS10A were characterized by the presence of mobile genetic elements. The presence of these elements within fosmids containing aromatic compound degradation genes suggests that the latter might have been acquired by different mechanisms of genetic transfer. The proposed relevance of mobile elements to the evolution and structure of catabolic pathways is supported by the genetic evidence that genes encoding various enzymatic steps are derived from existing single genes or whole operons, which are brought together by inter- and intra-cellular gene transfer mechanisms [69].

### Phylogenetic Affiliations of the Metagenomic Sequences

**Figure 6:**
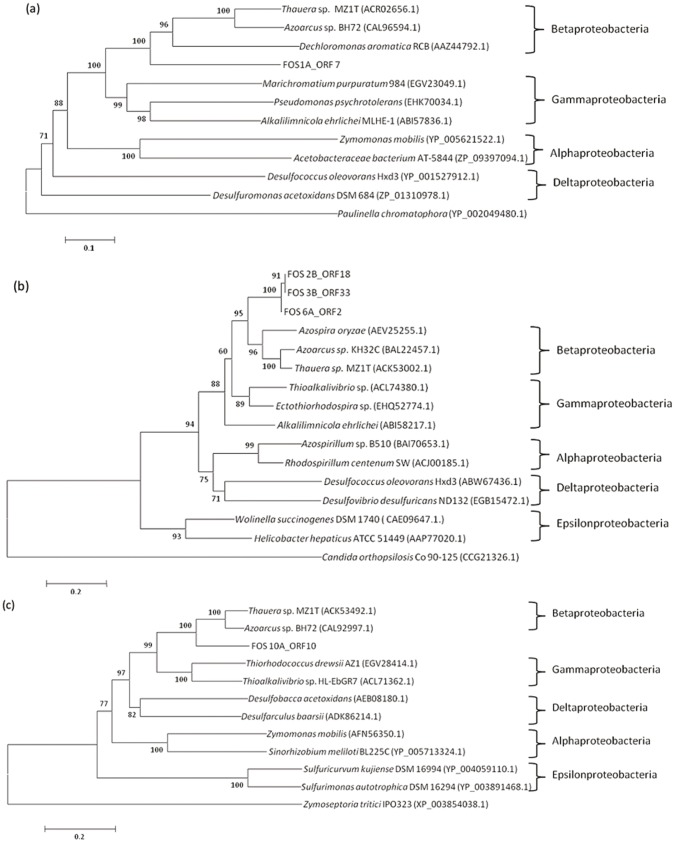
Phylogenetic analysis based on the deduced amino acid sequences of selected proteins in the metagenomic fosmid clones. (a) Primosomal protein N (COG L) of ORF 7 from fosmid clone FOS 1A, (b) ParB protein (COG K) of ORFs 18 from fosmid clone FOS 2B, ORF 33 from FOS 3B and ORF 2 from FOS 6A, and (c) arginine-tRNA ligase (COG J) of ORF 10 from fosmid clone FOS 10A. Bootstrap values (1000 replicate runs, shown as %) are listed. GenBank accession numbers are listed in parentheses after the species names.

In metagenomic analysis, phylogenetic assignment of metagenomic fragments is an important step that links the functional activities encoded by the DNA fragments to the phylogeny of an uncharacterized microorganism. All metagenomic clones were analyzed using the PhyloPythia software, and the results showed that all the metagenomic fragments were related to the phylum Proteobacteria. All fosmids were affiliated with the Betaproteobacteria class, except for fosmid FOS10A, which was related to the Gammaproteobacteria class (Table 2). In a second effort to determine the source organism of the DNA insert in each fosmid clone, phylogenetic trees were constructed using housekeeping genes, aiming to identify the closest relationships within the Proteobacteria phylum. For fosmid FOS1A, ORF 7 was selected for phylogenetic reconstruction (Figure 6a); this ORF encodes a primosomal protein (L COG category), which is a DNA helicase essential for DNA replication and synthesis of the RNA primer. For fosmids FOS2B, 3B and 6A, a phylogenetic tree was constructed using the ORFs encoding the ParB protein (K COG category) (Figure 6b), which is an enzyme required for efficient plasmid and chromosome partitioning. For fosmid 10A, the amino acid sequence of ORF 10 (Figure 6c), which encodes an arginine-tRNA ligase (J COG category) and its closest homologs, was used. Phylogenetic trees of the selected proteins were consistent with the phylogenetic classification at the class level inferred from the PhyloPythia analysis. The best hits annotated for each ORF in all clones (Supplementary tables S2 to S6) were predominantly related to the Betaproteobacteria class, even for FOS10A, which in contrast was more related to Gammaproteobacteria in the PhyloPythia test; however, a close relationship between the Gammaproteobacteria and Betaproteobacteria classes was observed in the phylogenetic tree (Figure 6c). These results suggest that all the metagenomic inserts could have originated from organisms closely related to the Betaproteobacteria class.

Members of the Betaproteobacteria class are able to aerobically degrade aromatic hydrocarbons, including chloroaromatic, nitroaromatic and aminoaromatic compounds. The most common genera include *Acidovorax* [70]–[72], *Burkholderia* [73]–[75] and *Polaromonas* [76], among others. Members of the Betaproteobacteria class can also anaerobically biodegrade aromatic compounds, especially under nitrate-reducing conditions [77]. This type of degradation is mainly performed by species of *Azoarcus* [78] and *Thauera* [79]. Furthermore, *Dechloromonas aromatica* RCB is able to degrade aromatic compounds under nitrate- or (per)chlorate-reducing conditions, as well as aerobically [80], [81].

Parales [77] proposed that the Betaproteobacteria may be more abundant or more efficient in terms of degradation in natural environments, but the fact that they do not grow as fast as some *Pseudomonas* isolates (Gammaproteobacteria) has probably delayed the understanding of their relevance in the environment. The Betaproteobacteria are widespread and have been detected in petroleum environments. A cultivation-independent study of the microbial community in the deep subsea-floor rock of a Brazilian oil reservoir [82] reported the predominance of clones belonging to the Betaproteobacteria class (53%), followed by the Alphaproteobacteria (25%). Vasconcellos et al. [10] and Silva et al. [83] used cultivation-dependent and independent studies to demonstrate that the class Betaproteobacteria constitutes a representative fraction of the total microbial diversity present in oil reservoir samples, encompassing the genera *Petrobacter*, *Thauera*, *Dechlorosoma* and *Hydrogenophilus*. In a recent study, Verde and co-workers [84] used degenerate primers to identify functional genes involved in aerobic and anaerobic hydrocarbon degradation in Brazilian petroleum reservoirs. The sequences found were highly similar to the ones present in microorganisms of the Betaproteobacteria group such as *Azoarcus* sp. *and Burkholderia* sp.

Based on our results, the sequences responsible for hydrocarbon degradation were related to microorganisms of the Betaproteobacteria class, which indicates the presence and active metabolism of these bacteria in oil reservoirs.

## Conclusions

The organization of hydrocarbon degradation-related genes in five fosmid clones, selected in a previous study, was unraveled. Data analysis showed that the complete hydrocarbon degradation pathways described in literature were absent in these clones. Instead, genes or gene subsets in novel arrangements were identified, suggesting that the observed aromatic compound degradation might occur through the concerted action of these fragmented pathways. These results indicate that there are significant differences between the degradation genes found in microbial communities derived from enrichments of oil reservoir samples and those that have been previously identified in bacteria isolated from contaminated and non-contaminated environments. These findings reinforce the potential of metagenomics to investigate the uncultivated majority and highlight the need for using petroleum reservoir samples to study biodegradation in this environment.

## Supporting Information

Table S1 **Number of Open Reading Frames of metagenomic fragments (FOS1A, FOS2B, FOS3B, FOS6A and FOS10A) assigned to different COG functional categories.**
(DOC)

Table S2 **Predicted and annotated ORFs of the fosmid FOS1A derived from a metagenomic library from petroleum reservoir.** ^a^References relate to UniProtKB (http://www.uniprot.org); [86]. ^b^COG database (http://www.ncbi.nlm.nih.gov/COG/; [19]). ^c^Hits were obtained from BLASTP comparison of predicted proteins from fosmids with UNIPROTKB database.
(DOC)

Table S3 **Predicted and annotated ORFs of the fosmid FOS2B derived from a metagenomic library from petroleum reservoir.** ^a^References relate to UniProtKB (http://www.uniprot.org); [86]. ^b^COG database (http://www.ncbi.nlm.nih.gov/COG/; [19]). ^c^Hits were obtained from BLASTP comparison of predicted proteins from fosmids with UNIPROTKB database.
(DOC)

Table S4 **Predicted and annotated ORFs of the fosmid FOS3B derived from a metagenomic library from petroleum reservoir.** ^a^References relate to UniProtKB (http://www.uniprot.org); [86]. ^b^COG database (http://www.ncbi.nlm.nih.gov/COG/; [19]). ^c^Hits were obtained from BLASTP comparison of predicted proteins from fosmids with UNIPROTKB database.
(DOC)

Table S5 **Predicted and annotated ORFs of the fosmid FOS6A derived from a metagenomic library from petroleum reservoir.** ^a^References relate to UniProtKB (http://www.uniprot.org); [86]. ^b^COG database (http://www.ncbi.nlm.nih.gov/COG/; [19]). ^c^Hits were obtained from BLASTP comparison of predicted proteins from fosmids with UNIPROTKB database.
(DOC)

Table S6 **Predicted and annotated ORFs of the fosmid FOS10A derived from a metagenomic library from petroleum reservoir.** ^a^References relate to UniProtKB (http://www.uniprot.org); [86]. ^b^COG database (http://www.ncbi.nlm.nih.gov/COG/; [19]). ^c^Hits were obtained from BLASTP comparison of predicted proteins from fosmids with UNIPROTKB database.
(DOC)
