# Supplementary material for: New Hydrocarbon Degradation Pathways in the Microbial Metagenome from Brazilian Petroleum Reservoirs
Source: PLoS One. 2014 Feb 26;9(2):e90087. doi: 10.1371/journal.pone.0090087 (PMC3935994; doi:10.1371/journal.pone.0090087)
Supplement: Table S1 — Number of Open Reading Frames of metagenomic fragments (FOS1A, FOS2B, FOS3B, FOS6A and FOS10A) assigned to different COG functional categories. (DOC) [file pone.0090087.s001.doc]

**Table S1.** Number of Open Reading Frames of metagenomic fragments (FOS1A, FOS2B, FOS3B, FOS6A and FOS10A) assigned to different COG functional categories.

| Class | Description | Fosmid clone ID | | | | | Total Number |
| --- | --- | --- | --- | --- | --- | --- | --- |
|  |  | FOS1A | FOS2B | FOS3B | FOSGA | FOS10A |  |
| Information Storage and Processing | |  |  |  |  |  |  |
| J | Translation | 1 |  |  |  | 2 | 3 |
| K | Transcription |  | 2 | 4 | 2 |  | 8 |
| L | Replication, recombination and repair | 2 |  |  | 2 | 3 | 7 |
| Cellular processes and Signaling | |  |  |  |  |  |  |
| D | Cell cycle control |  | 2 | 3 | 2 | 1 | 8 |
| T | Signal transduction mechanisms | 6 |  | 2 |  | 1 | 9 |
| M | Cell wall/membrane biogenesis | 2 | 2 | 1 | 2 |  | 7 |
| O | Posttranslational modification, protein turnover, chaperones | 1 | 1 | 1 |  | 2 | 5 |
| Metabolism | |  |  |  |  |  |  |
| C | Energy production and conversion | 6 | 11 | 10 | 4 |  | 31 |
| G | Carbohydrate transport and metabolism |  | 2 | 2 | 3 |  | 7 |
| E | Amino acid transport and metabolism | 3 | 5 | 5 | 3 | 2 | 18 |
| F | Nucleotide transport and metabolism | 1 |  |  |  |  | 1 |
| H | Coenzyme transport and metabolism | 1 | 1 | 2 | 1 | 1 | 6 |
| I | Lipid transport and metabolism |  | 2 | 2 | 2 | 1 | 7 |
| P | Inorganic ion transport and metabolism |  |  | 2 |  | 2 | 4 |
| Q | Secondary metabolites biosynthesis, transport and catabolism | 1 | 3 | 2 | 2 |  | 8 |
| Poorly characterized | |  |  |  |  |  |  |
| R | General function prediction | 3 | 3 | 2 | 3 | 2 | 13 |
| S | Function unknown | 1 | 2 | 2 | 2 | 1 | 8 |
| - | Not in COGs | 4 | 4 | 6 | 3 | 14 | 31 |
