# Supplementary material for: New Hydrocarbon Degradation Pathways in the Microbial Metagenome from Brazilian Petroleum Reservoirs
Source: PLoS One. 2014 Feb 26;9(2):e90087. doi: 10.1371/journal.pone.0090087 (PMC3935994; doi:10.1371/journal.pone.0090087)
Supplement: Table S2 — Predicted and annotated ORFs of the fosmid FOS1A derived from a metagenomic library from petroleum reservoir. aReferences relate to UniProtKB (http://www.uniprot.org); [86]. bCOG database (http://www.ncbi.nlm.nih.gov/COG/; [19]). cHits were obtained from BLASTP comparison of predicted proteins from fosmids with UNIPROTKB database. (DOC) [file pone.0090087.s002.doc]

Table S2. Predicted and annotated ORFs of the fosmid FOS1A derived from a metagenomic library from petroleum reservoir

| **ORF** | **Locus** | **Length (aminoacids)** | **UNIPROTKB references^a^** | **BLASTP hit used for annotation^c^** | | | | **Putative function** | **COG^b^** | **Taxonomical assignment**  **Phylum/Class** |
| --- | --- | --- | --- | --- | --- | --- | --- | --- | --- | --- |
|  |  |  |  | Gene name | Organism | E-value | Identity |  |  |  |
| **1** | 5_442 | 145 | C4K9F3 | speE | *Thauera* sp. (strain MZ1T) | 9.0×10^-54^ | 61% | Spermidine synthase | E | Proteobacteria/ b-proteobacteria |
| **2** | 439_1908 | 489 | A1KCP5 | azo3985 | *Azoarcus* sp. (strain BH72) | 1.0×10-136 | 51% | Sun protein | J | Proteobacteria/ b-proteobacteria |
| **3** | 1967_2482 | 171 | H1RSY1 | CTATCC11996_16749 | *Comamonas* *testosteroni* ATCC 11996 | 1.0×10^-14^ | 35% | Proline rich signal peptide protein | No related | Proteobacteria/ b-proteobacteria |
| **4** | 2479_4617 | 712 | Q5P4Q0 | AZOSEA15870 ebA2830 | *Aromatoleum* *aromaticum* (strain EbN1) | 0 | 51% | Sensory transduction histidine kinase | T | Proteobacteria/ b-proteobacteria |
| **5** | 4610_5854 | 414 | A1KCP2 | ntrX azo3982 | *Azoarcus* sp. (strain BH72) | 2.0×10^-90^ | 40% | Nitrogen assimilation regulatory protein | T | Proteobacteria/ b-proteobacteria |
| **6** | 5903_6973 | 356 | C4K9E7 | hemE | *Thauera* sp. (strain MZ1T) | 0 | 76% | Uroporphyrinogen decarboxylase | C | Proteobacteria/ b-proteobacteria |
| **7** | 6982_8967 | 661 | C4K9E5_THASP | Tmz1t_4079 | *Thauera* sp. (strain MZ1T) | 0 | 53% | Primosomal protein N' | L | Proteobacteria/ b-proteobacteria |
| **8** | 10149_9121 | 342 | A6Q1C3 | NIS_0168 | *Nitratiruptor* sp. (strain SB155-2) | 1.0×10-129 | 56% | Putative uncharacterized protein | S | Proteobacteria/ e-proteobacteria |
| **9** | 10361_10146 | 71 | G7UQW9 | DSC_11985 | *Ktedonobacter* *racemifer* DSM 44963 | 2.0×10^-11^ | 42% | Short-chain dehydrogenase/reductase SDR | QR | Chloroflexi |
| **10** | 10333_10602 | 89 | B8JHK9 | A2cp1_3389 | *Anaeromyxobacter* *dehalogenans* (strain 2CP-1) | 5.0×10^-31^ | 73% | Alcohol dehydrogenase zinc-binding domain protein | R | Proteobacteria/ d-proteobacteria |
| **11** | 10637_11314 | 225 | D4E6A0 | HMPREF0758_3700 | *Serratia odorifera* DSM 4582 | 1.0×10-123 | 75% | ThiJ/PfpI family protein | R | Proteobacteria/ g-proteobacteria |
| **12** | 11326_11631 | 101 | G4F5Z1 | HAL1_09562 | *Halomonas* sp. HAL1 | 3.0×10^-42^ | 70% | Putative uncharacterized protein | No related | Proteobacteria/ g-proteobacteria |
| **13** | 11665_12495 | 276 | Q88K03 | ecd PP_2490 | *Pseudomonas putida* (strain KT2440) | 1.0×10-110 | 62% | Flavoprotein  Nitroreductase | C | Proteobacteria/ g-proteobacteria |
| **14** | 12566_13957 | 463 | Q88K05 | PP_2488 | *Pseudomonas putida* (strain KT2440) | 0 | 80% | Succinic-semialdehyde dehydrogenase | C | Proteobacteria/ g-proteobacteria |
| **15** | 14045_15463 | 472 | G7LRS4 | BrE312_3551 | *Brenneria* sp. EniD312 | 0 | 68% | Aldehyde dehydrogenase | C | Proteobacteria/ g-proteobacteria |
| **16** | 15517_15927 | 136 | E3EY88 | EIO_0286 | *Ketogulonicigenium vulgare* (strain Y25) | 2.0×10^-65^ | 71% | OsmC-like protein | O | Proteobacteria/ a-proteobacteria |
| **17** | 15955_17010 | 351 | F8GXB3 | CNE_BB1p05630 | Cupriavidus necator (strain ATCC 43291) (*Ralstonia eutropha*) | 0 | 76% | Zinc dependen alcohol dehydrogenase | R | Proteobacteria/ b-proteobacteria |
| **18** | 17082_18095 | 337 | F6AHY1 | Psefu_2773 | *Pseudomonas fulva* (strain 12-X) | 0 | 77% | NADPH:quinone reductase | CR | Proteobacteria/ g-proteobacteria |
| **19** | 19081_18047 | 344 | Q1LNZ0 | tnp Rmet_1251 | *Ralstonia metallidurans* (strain CH34) | 1.0×10-153 | 68% | Transposase ISRme5 (Copy a, CMGI-2) | L | Proteobacteria/ b-proteobacteria |
| **20** | 19313_19759 | 148 | H0PZA7 | AZKH_2127 | *Azoarcus* sp. KH32C | 5.0×10^-45^ | 52% | Putative uncharacterized protein | No related | Proteobacteria/ b-proteobacteria |
| **21** | 21010_19754 | 418 | G4G165 | R2APBS1DRAFT_3672 | *Rhodanobacter* sp. 2APBS1 | 3.0×10^-45^ | 32% | Integral membrane sensor signal transduction histidine kinase | T | Proteobacteria/ g-proteobacteria |
| **22** | 21680_20988 | 230 | C1DDM2 | colR Avin_17800 | *Azotobacter vinelandii* (strain DJ) | 1.0×10^-82^ | 57% | Response regulator, transcriptional regulatory protein (Two-component), ColR | TK | Proteobacteria/ g-proteobacteria |
| **23** | 21896_23503 | 535 | A4BLM4 | NB231_15368 | *Nitrococcus mobilis* Nb-231 | 1.0×10-157 | 49% | Putative glycosyltransferase | M | Proteobacteria/ g-proteobacteria |
| **24** | 23509_24237 | 242 | Q3SIN6 | Tbd_1536 | *Thiobacillus denitrificans* (strain ATCC 25259) | 1.0×10^-83^ | 56% | Glycosyltransferase, group 2 family protein | M | Proteobacteria/ b-proteobacteria |
| **25** | 24234_24530 | 98 | A4BLM5 | NB231_15373 | *Nitrococcus mobilis* Nb-231 | 1.0×10^-38^ | 67% | Putative uncharacterized protein | No related | Proteobacteria/ g-proteobacteria |
| **26** | 26762_24591 | 723 | C6WYR6 | Mmol_0131 | *Methylotenera mobilis* (strain JLW8) | 0 | 47% | Diguanylate cyclase/phosphodiesterase | T | Proteobacteria/ b-proteobacteria |
| **27** | 27523_26759 | 254 | A1KBD6 | azo3526 | *Azoarcus* sp. (strain BH72) | 1.0×10-104 | 56% | Conserved hypothetical flavoprotein | C | Proteobacteria/ b-proteobacteria |
| **28** | 27662_28456 | 264 | H0Q123 | thyA AZKH_1296 | *Azoarcus* sp. KH32C | 1.0×10-164 | 79% | Thymidylate synthase | F | Proteobacteria/ b-proteobacteria |
| **29** | 28453_28947 | 164 | Q5P234 | dfrA AZOSEA25050 ebA4414 | *Aromatoleum aromaticum* (strain EbN1) | 7.0×10^-56^ | 58% | Dihydrofolate reductase | H | Proteobacteria/ b-proteobacteria |
| **30** | 31040_28944 | 698 | Q47GC0 | Daro_1362 | *Dechloromonas aromatica* (strain RCB) | 1.0×10-171 | 61% | Diguanylate cyclase/phosphodiesterase with PAS/PAC sensor(S) | T | Proteobacteria/ b-proteobacteria |
| **31** | 32370_31051 | 439 | Q47G42 | Daro_1440 | *Dechloromonas aromatica* (strain RCB) | 0 | 63% | Amino acid/amide ABC transporter substrate-binding protein, HAAT family | E | Proteobacteria/ b-proteobacteria |
| **32** | 34658_32424 | 744 | A1KAF9 | adiA azo3199 | *Azoarcus* sp. (strain BH72) | 0 | 83% | Orn/Lys/Arg decarboxylase | E | Proteobacteria/ b-proteobacteria |

^a^ References relate to UniProtKB (http://www.uniprot.org); [86]

^b^ COG database (<http://www.ncbi.nlm.nih.gov/COG/>; [19]).

^c^ Hits were obtained from BLASTP comparison of predicted proteins from fosmids with UNIPROTKB database.
