# Supplementary material for: New Hydrocarbon Degradation Pathways in the Microbial Metagenome from Brazilian Petroleum Reservoirs
Source: PLoS One. 2014 Feb 26;9(2):e90087. doi: 10.1371/journal.pone.0090087 (PMC3935994; doi:10.1371/journal.pone.0090087)
Supplement: Table S3 — Predicted and annotated ORFs of the fosmid FOS2B derived from a metagenomic library from petroleum reservoir. aReferences relate to UniProtKB (http://www.uniprot.org); [86]. bCOG database (http://www.ncbi.nlm.nih.gov/COG/; [19]). cHits were obtained from BLASTP comparison of predicted proteins from fosmids with UNIPROTKB database. (DOC) [file pone.0090087.s003.doc]

Table S3. Predicted and annotated ORFs of the fosmid FOS2B derived from a metagenomic library from petroleum reservoir

| **ORF** | **Locus** | **Length (aminoacids)** | **UNIPROTKB references^a^** | **BLASTP hit used for annotation^c^** | | | | **Putative function** | **COG^b^** | **Taxonomical assignment**  **Phylum/Class** |
| --- | --- | --- | --- | --- | --- | --- | --- | --- | --- | --- |
|  |  |  |  | Gene name | Organism | E-value | Identity |  |  |  |
| **1** | 2..163 | 395 | E3HP90 | AXYL_00107 | *Achromobacter xylosoxidans* (strain A8) | 5.0×10^-29^ | 91% | CoA-transferase family III family protein 1 | C | Proteobacteria/ b-proteobacteria |
| **2** | 181..1368 | 61 | C4ZIR1 | Tmz1t_0419 | *Thauera* sp. (strain MZ1T) | 0 | 86% | Acyl-CoA dehydrogenase domain protein | I | Proteobacteria/ b-proteobacteria |
| **3** | 1512..2423 | 311 | C4ZIR0 | Tmz1t_0418 | *Thauera* sp. (strain MZ1T) | 1.0×10-162 | 78% | Transcriptional regulator, LysR family | K | Proteobacteria/ b-proteobacteria |
| **4** | 2479..3198 | 239 | Q607F7 | MCA1803 | *Methylococcus capsulatus* (strain ATCC 33009) | 1.0×10-103 | 58% | Dienelactone hydrolase family protein | Q | Proteobacteria/ g-proteobacteria |
| **5** | 3202..4122 | 306 | D5T624 | lpa_02984 | *Legionella pneumophila* serogroup 1 (strain 2300) | 3.0×10^-43^ | 30% | ABC transport system periplasmic substrate binding protein | R | Proteobacteria/ g-proteobacteria |
| **6** | 4188..4778 | 196 | I3CFS1 | BegalDRAFT_1584 | *Beggiatoa alba* B18LD | 5.0×10^-13^ | 30% | ABC-type uncharacterized transport system, auxiliary component | S | Proteobacteria/ g-proteobacteria |
| **7** | 4775..5491 | 238 | H0Q2G0 | smuG AZKH_4546 | *Azoarcus* sp. KH32C | 1.0×10-122 | 70% | Single-strand selective monofunctional uracil DNA glycosylase | No related | Proteobacteria/ b-proteobacteria |
| **8** | 5532..6644 | 370 | Q4K4D9 | PFL_5836 | *Pseudomonas fluorescens* (strain Pf-5 ) | 7.0×10^-36^ | 51% | NLP/P60 family protein | M | Proteobacteria/ g-proteobacteria |
| **9** | 6733..7287 | 201 | E5APZ6 | RBRH_02409 | *Burkholderia rhizoxinica* (strain DSM 19002) | 2.0×10^-63^ | 58% | 3-polyprenyl-4-hydroxybenzoate decarboxylase ubiX | H | Proteobacteria/ b-proteobacteria |
| **10** | 7291..9042 | 583 | H0Q2F0 | ptsI AZKH_4536 | *Azoarcus* sp. KH32C | 0 | 64% | Phosphoenolpyruvate-protein phosphotransferase (ptsI) | G | Proteobacteria/ b-proteobacteria |
| **11** | 9047..9316 | 89 | C4ZL62 | Tmz1t_0468 | *Thauera* sp. (strain MZ1T) | 7.0×10^-41^ | 74% | Phosphotransferase system, phosphocarrier protein HPr | G | Proteobacteria/ b-proteobacteria |
| **12** | 9416..10216 | 260 | C7RQP5 | CAP2UW1_1059 | *Accumulibacter phosphatis* (strain UW-1) | 8.0×10^-81^ | 52% | Benzoate degradation ring-cleavage hydrolase | R | Proteobacteria/ b-proteobacteria |
| **13** | 10213..10671 | 152 | H0Q0W9 | AZKH_0270 | *Azoarcus* sp. KH32C | 2.0×10^-46^ | 52% | Putative uncharacterized protein | No related | Proteobacteria/ b-proteobacteria |
| **14** | 10700..11557 | 285 | H0Q0X0 | AZKH_0271 | *Azoarcus* sp. KH32C | 1.0×10-151 | 77% | SPFH domain, Band 7 family protein | O | Proteobacteria/ b-proteobacteria |
| **15** | 11626..13548 | 640 | H0PYM2 | gidA mnmG AZKH_0107 | *Azoarcus* sp. KH32C | 0 | 77% | tRNA uridine 5-carboxymethylaminomethyl modification enzyme MnmG | D | Proteobacteria/ b-proteobacteria |
| **16** | 13545..14213 | 222 | H0PYM3 | gidB rsmG AZKH_0108 | *Azoarcus* sp. KH32C | 1.0×10^-57^ | 49% | Ribosomal RNA small subunit methyltransferase G | D | Proteobacteria/ b-proteobacteria |
| **17** | 14213..15004 | 263 | B3R7M4 | parA1 RALTA_A3101 | *Cupriavidus taiwanensis* (strain R1) (*Ralstonia taiwanensis* (strain LMG 19424)) | 1.0×10-133 | 75% | Chromosome partitioning protein | M | Proteobacteria/ b-proteobacteria |
| **18** | 15004..15852 | 282 | H0PYM5 | spoOJ AZKH_0110 | *Azoarcus* sp. KH32C | 1.0×10-111 | 64% | ParB-like partition protein | K | Proteobacteria/ b-proteobacteria |
| **19** | 15857..17779 | 559 | F3LVJ5 | RBXJA2T_18618 | *Rubrivivax benzoatilyticus* JA2 | 0 | 55% | Sigma-54 dependent transcriptional regulator | Q | Proteobacteria/ b-proteobacteria |
| **20** | 18005..19186 | 393 | C5AMS6 | bglu_2g07050 | *Burkholderia glumae* (strain BGR1) | 0 | 82% | Iron-containing alcohol dehydrogenase | C | Proteobacteria/ b-proteobacteria |
| **21** | 19311..20831 | 506 | A4U358 | MGR_2501 | *Magnetospirillum gryphiswaldense* | 0 | 83% | Aldehyde dehydrogenase B | C | Proteobacteria/ a-proteobacteria |
| **22** | 20895..21950 | 351 | D5WPK1 | Btus_1549 | *Bacillus tusciae* (strain DSM 2912) | 0 | 74% | Alcohol dehydrogenase GroES domain protein | R | Firmicutes |
| **23** | 21972..22246 | 91 | C0N293 | MDMS009_82 | *Methylophaga thiooxydans* DMS010 | 3.0×10^-43^ | 69% | Putative uncharacterized protein | No related | Proteobacteria/ g-proteobacteria |
| **24** | 22290..22700 | 134 | G7UU90 | DSC_01630 | *Pseudoxanthomonas spadix* (strain BD-a59) | 2.0×10^-69^ | 83% | Putative uncharacterized protein | S | Proteobacteria/ g-proteobacteria |
| **25** | 22841..23638 | 265 | F9U2F5 | MarpuDRAFT_2386 | *Marichromatium purpuratum* 984 | 1.0×10-134 | 75% | Monosaccharide-transporting ATPase | E | Proteobacteria/ g-proteobacteria |
| **26** | 23628..25592 | 654 | F9U2F4 | MarpuDRAFT_2385 | *Marichromatium purpuratum* 984 | 0 | 63% | Long-chain-fatty-acid--CoA ligase | I | Proteobacteria/ g-proteobacteria |
| **27** | 25594..26481 | 295 | F9U2F3 | MarpuDRAFT_2384 | *Marichromatium purpuratum* 984 | 1.0×10-151 | 72% | ABC-type transporter, integral membrane subunit | E | Proteobacteria/ g-proteobacteria |
| **28** | 26485..27558 | 357 | F9U2F2 | MarpuDRAFT_2383 | *Marichromatium purpuratum* 984 | 0 | 71% | ABC-type transporter, integral membrane subunit | E | Proteobacteria/ g-proteobacteria |
| **29** | 27600..28850 | 416 | F9U2F1 | MarpuDRAFT_2382 | *Marichromatium purpuratum* 984 | 1.0×10-179 | 59% | Extracellular ligand-binding receptor | E | Proteobacteria/ g-proteobacteria |
| **30** | 28897..29718 | 273 | F9U2F0 | MarpuDRAFT_2381 | *Marichromatium purpuratum* 984 | 1.0×10-126 | 71% | ABC transporter related protein | E | Proteobacteria/ g-proteobacteria |
| **31** | 29761..30201 | 146 | H8MIT5 | COCOR_00820 | *Rhodospirillum photometricum* DSM 122 | 2e-42 | 55% | Phenylacetic acid degradation protein  (thioesterase) | Q | Proteobacteria/ a-proteobacteria |
| **32** | 30346..30702 | 118 | F7T0P2 | AXXA_12502 | *Achromobacter xylosoxidans* AXX-A | 1.0×10^-15^ | 40% | ATP synthase protein I | No related | Proteobacteria/ b-proteobacteria |
| **33** | 30712..31563 | 283 | ATP6 | atpB azo0153 | *Azoarcus* sp. (strain BH72) | 1.0×10-169 | 80% | ATP synthase subunit a | C | Proteobacteria/ b-proteobacteria |
| **34** | 31667..31912 | 81 | ATPL | atpE azo0154 | *Azoarcus* sp. (strain BH72) | 1.0×10^-56^ | 95% | ATP synthase subunit c | C | Proteobacteria/ b-proteobacteria |
| **35** | 31962..32435 | 157 | ATPF | atpF azo0155 | *Azoarcus* sp. (strain BH72) | 1.0×10^-78^ | 78% | ATP synthase subunit b | C | Proteobacteria/ b-proteobacteria |
| **36** | 32439..32981 | 180 | ATPD | atpH azo0156 | *Azoarcus* sp. (strain BH72) | 5.0×10^-64^ | 60% | ATP synthase subunit delta | C | Proteobacteria/ b-proteobacteria |
| **37** | 32992..34530 | 512 | H3K2D6 | atpA | *Pseudomonas hydrogenothermophila* | 0 | 89% | F-type ATP synthase alpha subunit | C | Proteobacteria/ g-proteobacteria |
| **38** | 34565..35434 | 289 | C4ZL51 | atpG | *Thauera* sp. (strain MZ1T) | 1.0×10-160 | 79% | ATP synthase gamma chain | C | Proteobacteria/ b-proteobacteria |
| **39** | 35464..36861 | 465 | H3K2D8 | atpD | *Pseudomonas* *hydrogenothermophila* | 0 | 93% | F-type ATP synthase beta subunit | C | Proteobacteria/ g-proteobacteria |
| **40** | 36948..37369 | 140 | H3K2D9 | atpC | *Pseudomonas hydrogenothermophila* | 7.0×10^-73^ | 81% | ATP synthase epsilon chain | C | Proteobacteria/ g-proteobacteria |

^a^ References relate to UniProtKB (http://www.uniprot.org); [86]

^b^ COG database (<http://www.ncbi.nlm.nih.gov/COG/>; [19]).

^c^ Hits were obtained from BLASTP comparison of predicted proteins from fosmids with UNIPROTKB database.
