# Supplementary material for: New Hydrocarbon Degradation Pathways in the Microbial Metagenome from Brazilian Petroleum Reservoirs
Source: PLoS One. 2014 Feb 26;9(2):e90087. doi: 10.1371/journal.pone.0090087 (PMC3935994; doi:10.1371/journal.pone.0090087)
Supplement: Table S4 — Predicted and annotated ORFs of the fosmid FOS3B derived from a metagenomic library from petroleum reservoir. aReferences relate to UniProtKB (http://www.uniprot.org); [86]. bCOG database (http://www.ncbi.nlm.nih.gov/COG/; [19]). cHits were obtained from BLASTP comparison of predicted proteins from fosmids with UNIPROTKB database. (DOC) [file pone.0090087.s004.doc]

Table S4. Predicted and annotated ORFs of the fosmid FOS3B derived from a metagenomic library from petroleum reservoir

| **ORF** | **Locus** | **Length (aminoacids)** | **UNIPROTKB references^a^** | **BLASTP hit used for annotation^c^** | | | | **Putative function** | **COG^b^** | **Taxonomical assignment**  **Phylum/Class** |
| --- | --- | --- | --- | --- | --- | --- | --- | --- | --- | --- |
|  |  |  |  | Gene name | Organism | E-value | Identity |  |  |  |
| **1** | 1_321 | 106 | A1K814 | **arsR** azo2352 | *Azoarcus* sp. (strain BH72) | 1.0×10^-31^ | 62% | Putative regulatory protein ArsR family | K | Proteobacteria/ b-proteobacteria |
| **2** | 324_749 | 141 | A1K815 | arsC2 azo2353 | *Azoarcus* sp. (strain BH72) | 2.0×10^-56^ | 67% | Putative arsenate reductase (Glutaredoxin) | K | Proteobacteria/ b-proteobacteria |
| **3** | 746_1792 | 348 | G8QGT8 | Dsui_1848 | *Azospira oryzae* (strain ATCC BAA-33) (*Dechlorosoma suillum*) | 0 | 78% | Arsenical-resistance protein | T | Proteobacteria/ b-proteobacteria |
| **4** | 1859_2242 | 127 | A9AS13 | Bmul_5669 BMULJ_05842 | *Burkholderia multivorans* (strain ATCC 17616) | 2.0×10^-44^ | 55% | Arsenical resistance operon trans-acting repressor ArsD | P | Proteobacteria/ b-proteobacteria |
| **5** | 2239_4053 | 604 | A1K818 | arsA azo2356 | *Azoarcus* sp. (strain BH72) | 0 | 71% | Arsenite-transporting ATPase | D | Proteobacteria/ b-proteobacteria |
| **6** | 4542_4105 | 145 | I3BTT5 | Thini_2212 | *Thiothrix nivea* DSM 5205 | 2.0×10^-65^ | 73% | HPP family protein | T | Proteobacteria/ g-proteobacteria |
| **7** | 4756_5376 | 206 | I3BTT5 | Thini_2213 | *Thiothrix nivea* DSM 5205 | 9.0×10^-51^ | 46% | Transcriptional regulator, TetR family | K | Proteobacteria/ g-proteobacteria |
| **8** | 5414_6148 | 244 | Q5P4D8 | ubiE AZOSEA17000 ebA3016 | *Aromatoleum aromaticum* (strain EbN1) | 1.0×10-127 | 72% | Ubiquinone/menaquinone biosynthesis methyltransferase ubiE | H | Proteobacteria/ b-proteobacteria |
| **9** | 6167_7039 | 290 | C4ZL55 | Tmz1t_0218 | *Thauera* sp. (strain MZ1T) | 2.0×10^-59^ | 42% | Import inner membrane translocase subunit Tim44 | No related | Proteobacteria/ b-proteobacteria |
| **10** | 7043_7630 | 195 | E5U865 | HMPREF0005_02512 | *Achromobacter xylosoxidans* C54 | 7.0×10^-21^ | 38% | Putative uncharacterized protein | No related | Proteobacteria/ b-proteobacteria |
| **11** | 8124_7684 | 146 | H3K2D9 | atpC | *Pseudomonas hydrogenothermophila* | 4.0×10^-76^ | 81% | ATP synthase epsilon chain | C | Proteobacteria/ g-proteobacteria |
| **12** | 9608_8211 | 465 | H3K2D8 | atpD | *Pseudomonas hydrogenothermophila* | 0 | 93% | F-type ATP synthase beta subunit | C | Proteobacteria/ g-proteobacteria |
| **13** | 10507_9638 | 289 | C4ZL51 | atpG | *Thauera* sp. (strain MZ1T) | 1.0×10-160 | 79% | ATP synthase gamma chain | C | Proteobacteria/ b-proteobacteria |
| **14** | 12080_10542 | 512 | H3K2D6 | atpA | *Pseudomonas hydrogenothermophila* | 0 | 89% | F-type ATP synthase alpha subunit | C | Proteobacteria/ g-proteobacteria |
| **15** | 12633_12091 | 180 | ATPD | atpH azo0156 | *Azoarcus* sp. (strain BH72) | 5.0×10^-64^ | 60% | ATP synthase subunit delta | C | Proteobacteria/ b-proteobacteria |
| **16** | 13110_12637 | 157 | H3K2D4 | atpF | *Pseudomonas hydrogenothermophila* | 4.0×10^-79^ | 77% | ATP synthase subunit b | C | Proteobacteria/ g-proteobacteria |
| **17** | 13405_13160 | 81 | ATPL | atpE azo0154 | *Azoarcus* sp. (strain BH72) | 1.0×10^-56^ | 95% | ATP synthase subunit c | C | Proteobacteria/ b-proteobacteria |
| **18** | 14360_13509 | 283 | ATP6 | atpB azo0153 | *Azoarcus* sp. (strain BH72) | 1.0×10-169 | 80% | ATP synthase subunit a | C | Proteobacteria/ b-proteobacteria |
| **19** | 14726_14370 | 118 | F7T0P2 | AXXA_12502 | *Achromobacter xylosoxidans* AXX-A | 1.0×10^-15^ | 40% | ATP synthase protein I | No related | Proteobacteria/ b-proteobacteria |
| **20** | 14871_15311 | 146 | H8MIT5 | COCOR_00820 | *Corallococcus coralloides* (strain ATCC 25202) (*Myxococcus coralloides*) | 1.0×10^-50^ | 59% | Thioesterase domain-containing protein | Q | Proteobacteria/ d-proteobacteria |
| **21** | 16175_15354 | 273 | F9U2F0 | MarpuDRAFT_2381 | *Marichromatium purpuratum* 984 | 1.0×10-126 | 71% | ABC transporter related protein | E | Proteobacteria/ g-proteobacteria |
| **22** | 17472_16222 | 416 | F9U2F1 | MarpuDRAFT_2382 | *Marichromatium purpuratum* 984 | 1.0×10-179 | 59% | Extracellular ligand-binding receptor | E | Proteobacteria/ g-proteobacteria |
| **23** | 18587_17514 | 357 | F9U2F2 | MarpuDRAFT_2383 | *Marichromatium purpuratum* 984 | 0 | 71% | ABC-type transporter, integral membrane subunit | E | Proteobacteria/ g-proteobacteria |
| **24** | 19478_18591 | 295 | F9U2F3 | MarpuDRAFT_2384 | *Marichromatium purpuratum* 984 | 1.0×10-151 | 72% | ABC-type transporter, integral membrane subunit | E | Proteobacteria/ g-proteobacteria |
| **25** | 21444_19480 | 654 | F9U2F4 | MarpuDRAFT_2385 | *Marichromatium purpuratum* 984 | 0 | 63% | Long-chain-fatty-acid--CoA ligase | I | Proteobacteria/ g-proteobacteria |
| **26** | 22231_21434 | 265 | F9U2F5 | MarpuDRAFT_2386 | *Marichromatium purpuratum* 984 | 1.0×10-134 | 75% | Monosaccharide-transporting ATPase | E | Proteobacteria/ g-proteobacteria |
| **27** | 22776_22372 | 134 | G7UU90 | DSC_01630 | *Pseudoxanthomonas spadix* (strain BD-a59) | 2.0×10^-69^ | 83% | Putative uncharacterized protein | S | Proteobacteria/ g-proteobacteria |
| **28** | 23101_22826 | 91 | C0N293 | MDMS009_82 | *Methylophaga thiooxydans* DMS010 | 3.0×10^-43^ | 69% | Putative uncharacterized protein | No related | Proteobacteria/ g-proteobacteria |
| **29** | 24177_23122 | 351 | D5WPK1 | Btus_1549 | *Bacillus tusciae* (strain DSM 2912) | 0 | 74% | Alcohol dehydrogenase GroES domain protein | R | Firmicutes |
| **30** | 25761_24241 | 506 | A4U358 | MGR_2501 | *Magnetospirillum gryphiswaldense* | 0 | 83% | Aldehyde dehydrogenase B | C | Proteobacteria/ a-proteobacteria |
| **31** | 27067_25886 | 393 | C5AMS6 | bglu_2g07050 | *Burkholderia glumae* (strain BGR1) | 0 | 82% | Iron-containing alcohol dehydrogenase | C | Proteobacteria/ b-proteobacteria |
| **32** | 27536_29215 | 559 | F3LVJ5 | RBXJA2T_18618 | *Rubrivivax benzoatilyticus* JA2 | 0 | 55% | Sigma-54 dependent transcriptional regulator | Q | Proteobacteria/ b-proteobacteria |
| **33** | 30068_29220 | 282 | G8QI95 | Dsui_0849 | *Azospira oryzae* (strain ATCC BAA-33) (*Dechlorosoma suillum*) | 1.0×10-112 | 64% | ParB-like partition protein | K | Proteobacteria/ b-proteobacteria |
| **34** | 30859_30068 | 263 | B3R7M4 | parA1 RALTA_A3101 | *Cupriavidus taiwanensis* (strain R1 / LMG 19424) (*Ralstonia taiwanensis* (strain LMG 19424)) | 1.0×10-133 | 75% | ATPase involved in chromosome partitioning, PARA protein | D | Proteobacteria/ b-proteobacteria |
| **35** | 31527_30859 | 222 | H0PYM3 | gidB rsmG AZKH_0108 | *Azoarcus* sp. KH32C | 1.0×10^-57^ | 49% | Ribosomal RNA small subunit methyltransferase G | M | Proteobacteria/ b-proteobacteria |
| **36** | 33446_31524 | 640 | H0PYM2 | gidA mnmG AZKH_0107 | *Azoarcus* sp. KH32C | 0 | 77% | tRNA uridine 5-carboxymethylaminomethyl modification enzyme MnmG | D | Proteobacteria/ b-proteobacteria |
| **37** | 34372_33515 | 285 | H0Q0X0 | AZKH_0271 | *Azoarcus* sp. KH32C | 1.0×10-151 | 77% | Band 7 family protein | O | Proteobacteria/ b-proteobacteria |
| **38** | 34859_34401 | 152 | H0Q0W9 | AZKH_0270 | *Azoarcus* sp. KH32C | 2.0×10^-46^ | 52% | Putative uncharacterized protein | No related | Proteobacteria/ b-proteobacteria |
| **39** | 35656_34856 | 266 | C7RQP5 | CAP2UW1_1059 | *Accumulibacter phosphatis* (strain UW-1) | 1.0×10^-80^ | 52% | Benzoate degradation ring-cleavage hydrolase | R | Proteobacteria/ b-proteobacteria |
| **40** | 35756_36025 | 89 | C4ZL62 | Tmz1t_0468 | *Thauera* sp. (strain MZ1T) | 7.0×10^-41^ | 74% | Phosphotransferase system, phosphocarrier protein | G | Proteobacteria/ b-proteobacteria |
| **41** | 36030_37781 | 583 | H0Q2F0 | ptsI AZKH_4536 | *Azoarcus* sp. KH32C | 0 | 64% | Phosphoenolpyruvate-protein phosphotransferase | G | Proteobacteria/ b-proteobacteria |
| **42** | 38390_37785 | 209 | E5APZ6 | RBRH_02409 | *Burkholderia rhizoxinica* (strain DSM 19002) | 2.0×10^-63^ | 58% | 3-polyprenyl-4-hydroxybenzoate decarboxylase ubiX (EC 4.1.1.-) | H | Proteobacteria/ b-proteobacteria |
| **43** | 38428_39057 | 239 | B7I117 | BCAH187_C0045 | *Bacillus cereus* (strain AH187) | 8.0×10^-16^ | 37% | NlpC/P60 family domain protein | No related | Firmicutes |
| **44** | 39071_39790 |  | Q9I391 | PA1631 | *Pseudomonas aeruginosa* (strain ATCC 15692) | 1.0×10-128 | 77% | Probable acyl-CoA dehydrogenase | I | Proteobacteria/ g-proteobacteria |
| **45** | 40675_39893 | 260 | A1K3R7 | azo0855 | *Azoarcus* sp. (strain BH72) | 1.0×10^-78^ | 51% | Uncharacterized protein | S | Proteobacteria/ b-proteobacteria |
| **46** | 41930_40776 | 384 | PPS_0739 | F8FT22_PSEPU | *Pseudomonas putida* S16 | 0 | 72% | Chromate transporter | P | Proteobacteria/ g-proteobacteria |

^a^ References relate to UniProtKB (http://www.uniprot.org); [86]

^b^ COG database (<http://www.ncbi.nlm.nih.gov/COG/>; [19]).

^c^ Hits were obtained from BLASTP comparison of predicted proteins from fosmids with UNIPROTKB database.
