# Supplementary material for: New Hydrocarbon Degradation Pathways in the Microbial Metagenome from Brazilian Petroleum Reservoirs
Source: PLoS One. 2014 Feb 26;9(2):e90087. doi: 10.1371/journal.pone.0090087 (PMC3935994; doi:10.1371/journal.pone.0090087)
Supplement: Table S5 — Predicted and annotated ORFs of the fosmid FOS6A derived from a metagenomic library from petroleum reservoir. aReferences relate to UniProtKB (http://www.uniprot.org); [86]. bCOG database (http://www.ncbi.nlm.nih.gov/COG/; [19]). cHits were obtained from BLASTP comparison of predicted proteins from fosmids with UNIPROTKB database. (DOC) [file pone.0090087.s005.doc]

Table S5. Predicted and annotated ORFs of the fosmid FOS6A derived from a metagenomic library from petroleum reservoir

| **ORF** | **Locus** | **Length (aminoacids)** | **UNIPROTKB references^a^** | **BLASTP hit used for annotation^c^** | | | | **Putative function** | **COG^b^** | **Taxonomical assignment**  **Phylum/Class** |
| --- | --- | --- | --- | --- | --- | --- | --- | --- | --- | --- |
|  |  |  |  | Gene name | Organism | E-value | Identity |  |  |  |
| **1** | 308_1981 | 557 | F3LVJ5 | RBXJA2T_18618 | *Rubrivivax benzoatilyticus* JA2 | 0 | 55% | Sigma-54 dependent transcriptional regulator | Q | Proteobacteria/ b-proteobacteria |
| **2** | 2973_2110 | 287 | G8QI95 | Dsui_0849 | *Azospira oryzae* (strain ATCC BAA-33) (*Dechlorosoma suillum*) | 1.0×10-112 | 64% | ParB-like partition protein | K | Proteobacteria/ b-proteobacteria |
| **3** | 3764_2973 | 263 | D6CP92 | parA THI_3796 | *Thiomonas* sp. (strain 3As) | 1.0×10-132 | 74% | Chromosome partitioning protein | D | Proteobacteria/ b-proteobacteria |
| **4** | 4432_3764 | 222 | H0PYM3 | gidB rsmG AZKH_0108 | *Azoarcus* sp. KH32C | 2.0×10^-59^ | 51% | Ribosomal RNA small subunit methyltransferase G | M | Proteobacteria/ b-proteobacteria |
| **5** | 6351_4429 | 640 | H0PYM2 | gidA mnmG AZKH_0107 | *Azoarcus* sp. KH32C | 0 | 77% | tRNA uridine 5-carboxymethylaminomethyl modification enzyme MnmG | D | Proteobacteria/ b-proteobacteria |
| **6** | 7277_6486 | 263 | C7RQP5 | CAP2UW1_1059 | *Accumulibacter phosphatis* (strain UW-1) | 9.0×10^-78^ | 51% | Alpha/beta hydrolase fold protein | R | Proteobacteria/ b-proteobacteria |
| **7** | 7402_7671 | 89 | C4ZL62 | Tmz1t_0468 | *Thauera* sp. (strain MZ1T) | 5.0×10^-41^ | 73% | Phosphotransferase system, phosphocarrier protein HPr | G | Proteobacteria/ b-proteobacteria |
| **8** | 7676_9427 | 583 | C4ZL61 | Tmz1t_0467 | *Thauera* sp. (strain MZ1T) | 0 | 64% | Phosphoenolpyruvate-protein phosphotransferase (ptsI) | G | Proteobacteria/ b-proteobacteria |
| **9** | 9990_9433 | 185 | E5APZ6 | RBRH_02409 | *Burkholderia rhizoxinica* (strain DSM 19002) | 2.0×10^-60^ | 57% | 3-polyprenyl-4-hydroxybenzoate decarboxylase ubiX | H | Proteobacteria/ b-proteobacteria |
| **10** | 10079_11161 | 360 | G8Q3L4 | PSF113_5538 | *Pseudomonas fluorescens* F113 | 3.0×10^-36^ | 50% | NlpC/P60 family domain protein | M | Proteobacteria/ g-proteobacteria |
| **11** | 11954_11235 | 239 | H0Q2G0 | smuG AZKH_4546 | *Azoarcus* sp. KH32C | 1.0×10-114 | 67% | Single-strand selective monofunctional uracil DNA glycosylase | No related | Proteobacteria/ b-proteobacteria |
| **12** | 12586_11951 | 211 | Q0A9D6 | Mlg_1202 | *Alkalilimnicola ehrlichei* (strain MLHE-1) | 1.0×10^-15^ | 28% | ABC-type uncharacterized transport system, auxiliary component | S | Proteobacteria/ b-proteobacteria |
| **13** | 13487_12591 | 298 | D5T624 | lpa_02984 | *Legionella pneumophila* serogroup 1 (strain 2300) | 2.0×10^-44^ | 30% | ABC transport system periplasmic substrate binding protein | R | Proteobacteria/ g-proteobacteria |
| **14** | 14233_13514 | 239 | Q607F7 | MCA1803 | *Methylococcus capsulatus* (strain ATCC 33009) | 1.0×10-104 | 58% | Dienelactone hydrolase family protein | Q | Proteobacteria/ g-proteobacteria |
| **15** | 15223_14288 | 311 | C4ZIR0 | Tmz1t_0418 | *Thauera* sp. (strain MZ1T) | 1.0×10-161 | 78% | Transcriptional regulator, LysR family | K | Proteobacteria/ b-proteobacteria |
| **16** | 15343_16530 | 395 | C4ZIR1 | Tmz1t_0419 | *Thauera* sp. (strain MZ1T) | 0 | 86% | Acyl-CoA dehydrogenase domain protein | I | Proteobacteria/ b-proteobacteria |
| **17** | 16548_17771 | 407 | H0PYF0 | coaT AZKH_0030 | *Azoarcus* sp. KH32C | 0 | 72% | CoA-transferase | C | Proteobacteria/ b-proteobacteria |
| **18** | 17954_19228 | 424 | Q7WJ54 | gdhA BB2646 | *Bordetella bronchiseptica (*strain ATCC BAA-588) (*Alcaligenes bronchisepticus*) | 0 | 69% | Glutamate dehydrogenase | E | Proteobacteria/ b-proteobacteria |
| **19** | 19245_20693 | 482 | C4ZIS0 | Tmz1t_0428 | *Thauera* sp. (strain MZ1T) | 0 | 84% | Succinic semialdehyde dehydrogenase | C | Proteobacteria/ b-proteobacteria |
| **20** | 20839_21417 | 192 | H2FX70 | GU3_12235 | *Oceanimonas* sp. (strain GK1) | 1.0×10-115 | 89% | Hydantoinase B/oxoprolinase | E | Proteobacteria/ g-proteobacteria |
| **21** | 21468_21944 | 158 | Q5P231 | istB AZOSEA16040 | *Aromatoleum aromaticum* (strain EbN1) | 5.0×10^-65^ | 75% | Transposition helper protein | No related | Proteobacteria/ b-proteobacteria |
| **22** | 21941_23023 | 360 | Q5P3V1 | istA AZOSEA16050 | *Aromatoleum aromaticum* (strain EbN1) | 0 | 84% | Transposase | L | Proteobacteria/ b-proteobacteria |
| **23** | 23123_24328 | 401 | D6ZXD4 | BLJ_0086 | *Bifidobacterium longum* subsp. longum (strain JDM301) | 3.0×10^-31^ | 27% | Major facilitator superfamily MFS_1 | GEPR | Actinobacteria |
| **24** | 25513_24356 | 385 | Q1LNZ0 | tnp Rmet_1251 | *Ralstonia metallidurans* (strain CH34) | 1.0×10-135 | 60% | Transposase ISRme5 (Copy a, CMGI-2) | L | Proteobacteria/ b-proteobacteria |
| **25** | 25830_27011 | 393 | C5AMS6 | bglu_2g07050 | *Burkholderia glumae* (strain BGR1) | 0 | 82% | Iron-containing alcohol dehydrogenase | C | Proteobacteria/ b-proteobacteria |
| **26** | 27136_28656 | 506 | A4U358 | MGR_2501 | *Magnetospirillum gryphiswaldense* | 0 | 84% | Aldehyde dehydrogenase B | C | Proteobacteria/ a-proteobacteria |
| **27** | 28717_29772 | 351 | D5WPK1 | Btus_1549 | *Bacillus tusciae* (strain DSM 2912) | 0 | 75% | Alcohol dehydrogenase GroES domain protein | R | Firmicutes |
| **28** | 29829_30230 | 133 | F0BKU8 | XVE_4915 | *Xanthomonas vesicatoria* ATCC 35937 | 1.0×10^-69^ | 76% | Putative uncharacterized protein | S | Proteobacteria/ g-proteobacteria |
| **29** | 30371_31168 | 265 | F9U2F5 | MarpuDRAFT_2386 | *Marichromatium purpuratum* 984 | 1.0×10-135 | 75% | Monosaccharide-transporting ATPase | E | Proteobacteria/ g-proteobacteria |
| **30** | 31158_32837 | 559 | F9U2F4 | MarpuDRAFT_2385 | *Marichromatium purpuratum* 984 | 0 | 64% | Long-chain-fatty-acid--CoA ligase | I | Proteobacteria/ g-proteobacteria |
| **31** | 32846_32986 | 46 | Q2KTV6 | hyuA BAV3294 | *Bordetella avium* (strain 197N) | 1.0×10-147 | 76% | Hydantoin hydantoinase A | No related | Proteobacteria/ b-proteobacteria |

^a^ References relate to UniProtKB (http://www.uniprot.org); [86]

^b^ COG database (<http://www.ncbi.nlm.nih.gov/COG/>; [19]).

^c^ Hits were obtained from BLASTP comparison of predicted proteins from fosmids with UNIPROTKB database.
