# Supplementary material for: New Hydrocarbon Degradation Pathways in the Microbial Metagenome from Brazilian Petroleum Reservoirs
Source: PLoS One. 2014 Feb 26;9(2):e90087. doi: 10.1371/journal.pone.0090087 (PMC3935994; doi:10.1371/journal.pone.0090087)
Supplement: Table S6 — Predicted and annotated ORFs of the fosmid FOS10A derived from a metagenomic library from petroleum reservoir. aReferences relate to UniProtKB (http://www.uniprot.org); [86]. bCOG database (http://www.ncbi.nlm.nih.gov/COG/; [19]). cHits were obtained from BLASTP comparison of predicted proteins from fosmids with UNIPROTKB database. (DOC) [file pone.0090087.s006.doc]

Table S6. Predicted and annotated ORFs of the fosmid FOS10A derived from a metagenomic library from petroleum reservoir

| **ORF** | **Locus** | **Length (aminoacids)** | **UNIPROTKB references^a^** | **BLASTP hit used for annotation^c^** | | | | **Putative function** | **COG^b^** | **Taxonomical assignment**  **Phylum/Class** |
| --- | --- | --- | --- | --- | --- | --- | --- | --- | --- | --- |
|  |  |  |  | Gene name | Organism | E-value | Identity |  |  |  |
| **1** | 184_735 | 183 | A4XYT9 | Pmen_3757 | *Pseudomonas mendocina* (strain ymp) | 1.0×10^-65^ | 59% | CRISPR-associated protein, Cas5e family | No related | Proteobacteria/ g-proteobacteria |
| **2** | 720_1445 | 241 | CSE3 | cse3 TTHB192 | *Thermus thermophilus* (strain HB8) | 8.0×10^-37^ | 38% | CRISPR-associated endoribonuclease Cse3 | No related | Deinococcus-Thermus |
| **3** | 1456_2376 | 306 | D8PDH8 | cas1 NIDE1548 | Candidatus *Nitrospira defluvii* | 1.0×10-169 | 77% | CRISPR-associated protein Cas1 | No related | Nitrospirae |
| **4** | 2405_2671 | 88 | D8PDH9 | cas2 NIDE1549 | Candidatus *Nitrospira defluvii* | 2.0×10^-39^ | 80% | CRISPR-associated protein Cas2 | No related | Proteobacteria/ g-proteobacteria |
| **5** | 2777_2899 | 40 | B6WPR5 | DESPIG_00030 | *Desulfovibrio piger* ATCC 29098 | 5.0×10^-4^ | 56% | Putative uncharacterized protein | No related | Proteobacteria/ d-proteobacteria |
| **6** | 2910_3182 | 90 | H5WFZ9 | RSK60_750006 | *Ralstonia solanacearum* K60-1 | 1.0×10^-11^ | 50% | Putative uncharacterized protein | No related | Proteobacteria/ b-proteobacteria |
| **7** | 4929_5453 | 174 | B4Y375 | tnpA | *Thauera* sp. B4 | 2.0×10^-80^ | 68% | Transposase | L | Proteobacteria/ b-proteobacteria |
| **8** | 5572_5805 | 77 | G8QH35 | dsbD Dsui_0718 | *Azospira oryzae* (strain ATCC BAA-33 ) (*Dechlorosoma suillum*) | 2.0×10^-3^ | 45% | Thiol:disulfide interchange protein DsbD | No related | Proteobacteria/ b-proteobacteria |
| **9** | 7413_5887 | 508 | H0PRJ8 | ubiB AZKH_0494 | *Azoarcus* sp. KH32C | 0 | 73% | Putative ubiquinone biosynthesis protein | R | Proteobacteria/ b-proteobacteria |
| **10** | 7519_9306 | 595 | C4ZIX9 | argS | *Thauera* sp. (strain MZ1T) | 0 | 71% | Arginine--tRNA ligase | J | Proteobacteria/ b-proteobacteria |
| **11** | 9314_9949 | 211 | C4ZIY0 | Tmz1t_0721 | *Thauera* sp. (strain MZ1T) | 3.0×10^-39^ | 37% | Sporulation domain protein | D | Proteobacteria/ b-proteobacteria |
| **12** | 9965_10603 | 212 | A1K2E4 | dsbA azo0382 | *Azoarcus* sp. (strain BH72) | 1.0×10^-66^ | 54% | Thiol:disulfide interchange protein DsbA | OC | Proteobacteria/ b-proteobacteria |
| **13** | 10705_11967 | 420 | Q7NTI2 | tyrS CV_3072 | *Chromobacterium violaceum* (strain ATCC 12472) | 0 | 70% | Tyrosine--tRNA ligase | J | Proteobacteria/ b-proteobacteria |
| **14** | 11964_13460 | 498 | Q7NQY5 | CV_4000 | *Chromobacterium violaceum* (strain ATCC 12472) | 0 | 63% | Probable competence protein ComM | O | Proteobacteria/ b-proteobacteria |
| **15** | 13472_14116 | 214 | C4KDL3 | pdxH | *Thauera* sp. (strain MZ1T) | 1.0×10-105 | 69% | Pyridoxine/pyridoxamine 5'-phosphate oxidase | H | Proteobacteria/ b-proteobacteria |
| **16** | 16131_14134 | 665 | B8GTY7 | Tgr7_0171 | *Thioalkalivibrio* sp. (strain HL-EbGR7) | 3.0×10^-75^ | 37% | Diguanylate cyclase/phosphodiesterase with PAS/PAC sensor(S) | T | Proteobacteria/ g-proteobacteria |
| **17** | 17000_16128 | 290 | Q2W1L1 | amb3460 | *Magnetospirillum magneticum* (strain AMB-1) | 2.0×10^-28^ | 30% | ABC-type phosphate/phosphonate transport system, periplasmic component | P | Proteobacteria/ a-proteobacteria |
| **18** | 17673_17050 | 207 | F7S0W4 | A28LD_2173 | *Idiomarina* sp. A28L | 1.0×10^-72^ | 51% | Carbonic anhydrase | P | Proteobacteria/ g-proteobacteria |
| **19** | 17762_18664 | 300 | A1K272 | paaJ1 azo0310 | *Azoarcus* sp. (strain BH72) | 1.0×10^-93^ | 71% | Probable beta-ketoadipyl CoA thiolase | I | Proteobacteria/ b-proteobacteria |
| **20** | 18791_19516 | 241 | H0BV57 | KYG_06294 | *Acidovorax* sp. NO-1 | 1.0×10-122 | 75% | Extracellular ligand-binding receptor | E | Proteobacteria/ b-proteobacteria |
| **21** | 19556_20188 | 210 | B3E0V3 | Minf_2376 | *Methylacidiphilum infernorum* (isolate V4) | 1.0×10-121 | 84% | Transposon IS605 OrfA, integrase-resolvase | L | Verrumicrobia |
| **22** | 20185_21381 | 398 | H1SBA4 | OR16_27257 | *Cupriavidus basilensis* OR16 | 0 | 80% | Transposase OrfB | L | Proteobacteria/ b-proteobacteria |
| **23** | 21414_22169 | 251 | C4ZP82 | Tmz1t_1520 | *Thauera* sp. (strain MZ1T) | 1.0×10-134 | 79% | ABC transporter related protein | E | Proteobacteria/ b-proteobacteria |
| **24** | 22189_22770 | 193 | C4ZP83 | Tmz1t_1521 | *Thauera* sp. (strain MZ1T) | 3.0×10^-63^ | 51% | Hemerythrin HHE cation binding domain protein | No related | Proteobacteria/ b-proteobacteria |
| **25** | 22899_23216 | 105 | F7SLP2 | GME_07061 | *Halomonas boliviensis* LC1 | 1.0×10^-29^ | 53% | Inner membrane protein yqjE | No related | Proteobacteria/ g-proteobacteria |
| **26** | 23237_23611 | 124 | E1VA80 | HELO_2042 | *Halomonas elongata* (strain ATCC 33173) | 7.0×10^-33^ | 58% | Putative uncharacterized protein | No related | Proteobacteria/ g-proteobacteria |
| **27** | 23611_23931 | 106 | Q1QXL2 | Csal_1443 | *Chromohalobacter salexigens* (strain DSM 3043) | 3.0×10^-10^ | 42% | Putative uncharacterized protein | No related | Proteobacteria/ g-proteobacteria |
| **28** | 23989_24228 | 79 | H0Q099 | AZKH_4420 | *Azoarcus* sp. KH32C | 1.0×10^-27^ | 60% | Putative uncharacterized protein | S | Proteobacteria/ b-proteobacteria |
| **29** | 24398_27019 | 873 | H7F011 | PstZobell_18110 | *Pseudomonas stutzeri* ATCC 14405 | 0 | 50% | CRISPR-associated helicase Cas3 family protein | R | Proteobacteria/ g-proteobacteria |
| **30** | 27016_28518 | 500 | H7F010 | PstZobell_18105 | *Pseudomonas stutzeri* ATCC 14405 | 1.0×10-123 | 45% | CRISPR-associated Cse1 family protein | No related | Proteobacteria/ g-proteobacteria |
| **31** | 28568_29107 | 179 | I0HLZ5 | cse2 RGE_06870 | *Rubrivivax gelatinosus* (strain NBRC 100245) | 4.0×10^-40^ | 46% | CRISPR-associated protein, Cse2 family | No related | Proteobacteria/ b-proteobacteria |
| **32** | 29133_30269 | 378 | C1DSH8 | Avin_17200 | *Azotobacter vinelandii* (strain DJ) | 1.0×10-142 | 57% | CRISPR-associated protein, CT1975 | No related | Proteobacteria/ g-proteobacteria |

^a^ References relate to UniProtKB (http://www.uniprot.org); [86]

^b^ COG database (<http://www.ncbi.nlm.nih.gov/COG/>; [19]).

^c^ Hits were obtained from BLASTP comparison of predicted proteins from fosmids with UNIPROTKB database.
